# Ultra-High-Frequency-Dielectrophoresis Microfluidic Biosensor to Detect the Transformation Potential of Extracellular Vesicles Derived from Cancer Stem Cells

**DOI:** 10.3390/bios16010002

**Published:** 2025-12-19

**Authors:** Elodie Barthout, Elisa Lambert, Stéphanie Durand, Céline Hervieu, Léa Ikhlef, Sofiane Saada, Rémi Manczak, Julie Pannequin, Arnaud Pothier, Claire Dalmay, Fabrice Lalloué, Muriel Mathonnet, Barbara Bessette

**Affiliations:** 1 UMR INSERM 1308 CAPTuR, University of Limoges, 87025 Limoges, France; elodie.barthout@gu.se (E.B.); stephanie.durand@unilim.fr (S.D.); fabrice.lalloue@unilim.fr (F.L.); muriel.mathonnet@unilim.fr (M.M.); 2 UMR CNRS 7252 XLIM, University of Limoges, 87060 Limoges, France; elisa.lambert@cmf.hu (E.L.); arnaud.pothier@unilim.fr (A.P.); claire.dalmay@unilim.fr (C.D.); 3 UMR 5203 INSERM/CNRS, Institute of Functional Genomics, University of Montpellier, 34094 Montpellier, France; julie.pannequin@igf.cnrs.fr; 4 Department of General, Endocrine and Digestive Surgery, University Hospital of Limoges, 87025 Limoges, France

**Keywords:** colorectal cancer, cancer stem cells, extracellular vesicles, high frequency dielectrophoresis, microfluidic, lab-on-a-chip, point-of-care testing, biomedical applications

## Abstract

Cancer stem cells (CSCs) remain challenging to isolate and characterize because of their plastic phenotype. To overcome this issue, we used a microfluidic lab-on-a-chip analysis approach based on ultra-high frequency dielectophoresis (UHF-DEP) to measure the dielectrophoretic signature of colorectal cancer cells. We demonstrated that CSCs exhibit a distinct and lower frequency signature than differentiated cancer cells. Extracellular vesicles (EVs) released by tumor cells are implicated in tumor progression and metastasis. As CSC-derived EVs carry a more aggressive cargo, we hypothesized that treating differentiated colorectal cancer cells with these vesicles might affect their phenotype which would be detected by our lab on a chip. Indeed, the dielectrophoretic signature of cells treated with those EVs was altered in comparison to untreated cells, even in cases where no detectable biological changes were observed. Compared to conventional approaches using biomarkers to characterize CSCs, this UHF-DEP lab on a chip is a label-free method providing rapid and relevant results. Such a method could be useful in the clinic for the early detection of CSCs in the tumor mass, as well as for monitoring CSC-derived EVs in the bloodstream in order to study responses to therapy and prevent relapses.

## 1. Introduction

Colorectal cancer (CRC) is the second most diagnosed cancer in women and the third in men, yet mortality in women is 25% lower than in men. Industrialized countries have the highest CRC rates but these appear to be stabilizing or even declining due to screening campaigns and increased use of colonoscopy. Despite this, CRC is the fourth most deadly cancer worldwide with nearly 900,000 deaths per year [1]. Furthermore, nearly 25% of newly diagnosed CRCs present distant metastases [2] and 50% of patients will develop metastases during their lifetime [3,4]. Over the last decades, the diagnosis and care of CRC patients have considerably improved but relapses still occur and may be related to therapeutic resistance and/or the presence of a microscopic residual disease (MRD).

A particular subpopulation within the heterogeneous tumor mass, known as cancer stem cells (CSCs) or tumor-initiating cells, is responsible for both phenomena [5,6]. These are highly plastic undifferentiated cells resistant to conventional therapies due to their stemness properties. Regarding CRC, there is a direct correlation between a high number of undifferentiated cancer cells and a high risk of relapse [4,7].

Inside the stem cell niche, CSCs communicate with their microenvironment by secreting extracellular vesicles (EVs). These are lipid membrane-based particles ranging in size from 50 to 150 nm secreted by various cell types and found in all body fluids, but usually, tumor cells release more EVs than their healthy counterparts [8,9]. EVs are mediators of cell–cell communication by the exchange of their cargo, consisting of nucleic acids, proteins and lipids [8], thus they play a crucial role in tumor development [2,10], drug resistance [2,10] and transfer of aggressiveness [2,11,12] in solid cancers. We previously demonstrated that EVs can induce an aggressive phenotype by transferring neurotrophin receptors (TrkB) to YKL-40-inactivated glioblastoma (GBM) cells that have lost their aggressive potential [11]. Similarly, in GBM, EVs can transfer EGFRvIII, the truncated EGFR receptor associated with tumor progression [13]. In CRC, numerous studies have highlighted the role of EVs in the transfer of aggressiveness. EVs derived from CRC cell lines have been shown to deliver mRNAs, microRNAs (miRNAs) and natural antisense RNAs to other solid cancer cell lines to control their gene expression [14] or promote cell migration [15]. Colon cancer-derived EVs can stimulate tumor angiogenesis by activating endothelial cells [16], promoting acquired resistance to 5-fluorouracile (5-FU) [17] and thus enabling tumor growth and metastasis [12]. EVs can also affect the antitumor immune response by inhibiting the activation of macrophages [18], blocking T-cell proliferation and promoting regulator T-cell expansion [19]. In summary, EVs are considered as an important source of circulating tumor biomarkers. Therefore, they could represent a relevant strategy to remotely monitor the crosstalk between CSCs and their microenvironment and thus predict drug resistance, tumor progression and the risk of relapse.

**Figure 1:**
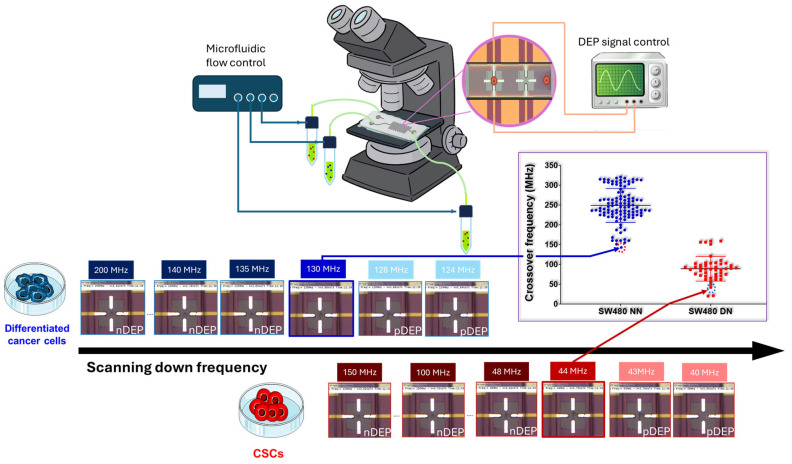
Principle of high frequency crossover frequency measurement.

Isolating and characterizing CSCs is challenging as they represent less than 10% of the tumor mass [20]. Their characterization involves assessing their functional properties and using biomarkers that are also shared with normal stem cells. All these techniques are time consuming and quite expensive. Thus, over the last few years, we developed a new label-free approach based on the biophysical properties of cells [21]. This technique measures the crossover frequency of single cells using the dielectrophoresis principle (DEP) [22,23]. Unlike standard DEP studies [24,25,26], our investigation focuses on the cell’s dielectric behavior in the tens to few hundreds of MHz range. The crucial point is that using high frequencies allows the electrical signal to ‘see’ inside the cell [27,28], whereas lower frequencies would primarily measure only the cell membrane’s properties [23]. To do so, a quadrupole electrode sensor is implemented in a microfluidic channel as illustrated in Figure 1. It allows for the electromanipulation of individual cells in suspension thanks to a continuous wave megahertz frequency electric field generated with a high frequency generator. Within the microfluidic channel, the cell’s movement is monitored visually as the frequency of the electrical generator is swept. The crossover frequency is determined by the onset of an attractive effect at one of the four sensor electrodes. We have previously demonstrated the discriminating potential of crossover frequency between CSCs and differentiated cancer cells from brain tumors [28,29] indicating that our UHF-DEP lab-on-chip sensor could be an interesting tool for CSC identification in clinical routines.

In this study, we used this biosensor to investigate whether colorectal CSCs and differentiated cancer cells exhibit a different crossover frequency, as previously observed in brain tumors [28,29]. In addition, we evaluated the possibility of monitoring phenotypic changes caused by EVs intake by measuring the biophysical properties of treated cells. Here, we report that colorectal CSCs exhibit a lower crossover frequency (lower than 100 MHz) than their differentiated counterparts, in both cell lines and primary cell cultures. In addition, this technique was able to detect phenotypic changes caused by EV intake that might be missed using conventional biological approaches. This label-free method allows for the biophysical characterization of cells and therefore could be useful to detect the presence of CSCs in a tumor mass in order to adapt the care of CRC patients.

## 2. Materials and Methods

### 2.1. Cell Culture

#### 2.1.1. Cell Lines and Patient-Derived Primary Cells

SW480 and SW620 cell lines were purchased from the American Type Culture Collection (ATCC/LGC Promochem, Molsheim, France) and grown in RPMI 1640 GlutaMAX^TM^ (Gibco^TM^, Thermo Fisher, Waltham, MA, USA) medium supplemented with 10% FBS, 1% sodium pyruvate and 1% of penicillin/streptomycin (Thermo Fisher).

Two primary cultures from patients (CPP) were kindly provided by Julie Pannequin’s team from the Institute of Functional Genomics (Univ. Montpellier, France) after informed consent of patients (Material Transfer Agreement CNRS 190287). CPP were grown in DMEM GlutaMAX^TM^ medium supplemented with 10% FBS and 1% penicillin/streptomycin (Thermo Fisher). Cell culture with FBS will be referred to “normal medium” (NM). All cell types were cultured at 37 °C in a humidified atmosphere of 5% CO_2_ and 95% air.

#### 2.1.2. Cancer Stem Cell Enrichment

To enrich the cell population in CSCs, both cell lines and CPP were cultured and maintained for at least a week in a “defined medium” (DM) consisting of FBS-free DMEM/F-12 (Thermo Fisher) medium supplemented with 5 µg/mL of insulin, N-2 supplement (1X), 1% of penicillin/streptomycin (Thermo Fisher), 20 ng/mL of EGF, and 20 ng/mL of bFGF (MACS Miltenyi Biotec, Bergisch Gladbach, Germany).

#### 2.1.3. Cell Treatment with Extracellular Vesicles

To test a transfer of aggressiveness, cells were seeded in a 6-well plate at 0.25 × 10^6^ cells and treated with EVs released from 1 × 10^6^ cells (NM or DM cultured cells) resuspended in an “exo-free” medium. To obtain an exo-free medium, FBS was depleted in EVs by ultracentrifugation at 120,000× *g* for 16 h. Cells were treated for 24 h or 72 h once or twice with the same amount of EVs each time.

### 2.2. Extracellular Vesicles

#### 2.2.1. Isolation

Cells were seeded in a T75 flask at 1 × 10^6^ cells with 10 mL of medium and cultured for 48 h before harvesting the supernatant. EVs were pelleted by ultracentrifugation of the supernatant at 120,000× *g* for 90 min and washed with phosphate-buffered saline (PBS, Thermo Fisher) under the same conditions. Finally, EVs were either resuspended in PBS for nanoparticle tracking analysis (NTA) in culture medium to treat cells or in RIPA buffer for Western blot analysis.

#### 2.2.2. Nanoparticle Tracking Analysis (NTA)

NTA was performed using NanoSight NS300 (Malvern Panalytical Ltd., Malvern, UK) with specific parameters according to the manufacturer’s user manual (NanoSight NS300 User Manual, MAN0541-01-EN-00, 2017). Captures and analysis were achieved by using the built-in NanoSight Software NTA3.3.301 (Malvern Panalytical Ltd.). The camera level was set at 14 and the detection threshold was fixed at 5. Samples were diluted in PBS and the concentration adjusted until observing a particle/frame rate around 50 (30–100 particles/frame). For each measurement, five consecutive 60 s videos were recorded under the following conditions: cell temperature—25 °C; syringe speed—22 μL/s (100 a.u.). Particles (EVs) were detected using a 488 nm laser (blue) and a scientific CMOS camera. Among the information given by the software, the following were studied: mode (i.e., the most represented EV size population), the percentage of EVs between 50 and 150 nm and particles/mL. Using the last one and the number of cells collected, the EV/cell ratio was calculated.

### 2.3. RNA Extraction and RT-qPCR

Total RNA was extracted using RNeasy kit (Qiagen, Hilden, Germany) according to the manufacturer’s instructions and quantified by NanoDrop 2000 (Thermo Fischer, Waltham, MA, USA). For qRT-PCR analyses, 2 µg of RNA was reverse-transcribed into cDNA using the cDNA Archive kit (Applied Biosystems, Waltham, MA, USA). Quantitative gene expression was performed using SensiFAST Probe Hi-ROX kit (Bioline, London, UK) on QuantStudio 5 (Thermo Fischer), and stemness-related genes (*PROM1*, *NANOG*, *SOX2*, *POU5F1*, *BMI1*, *LGR5*) were analyzed. Results were normalized to *GADPH* and *HPRT1* expressions and analyzed using the ∆∆Ct method (control condition defined as 1).

### 2.4. Western Blot

After collection, EVs were resuspended and lyzed in RIPA buffer (50 mM Tris-HCl, pH 7.4, 1% NP-40, 0.5% Na-deoxycholate, 0.1% SDS, 150 mM NaCl, 2 mM EDTA, 50 mM NaF) supplemented extemporaneously with a cocktail of protease inhibitors (Roche, Bâle, Switzerland). Samples were separated on a 12% SDS-polyacrylamide gel electrophoresis, transferred to polyvinylidene difluoride (PVDF) membrane, blocked for 1 h in 5% (*w*/*v*) bovine serum albumin (BSA) in PBS and probed overnight at 4 °C with EVs-specific primary antibodies (Alix, Hsc70 and CD9). Membranes were then incubated with the proper secondary antibodies for 1 h and proteins revealed by Immobilon ECL Ultra (Merck Milli-pore, Burlington, MA, USA) using a G-box imager with GeneSnap software (V1.6.1 Syngene).

### 2.5. Flow Cytometry

#### 2.5.1. Stemness Markers

Differentiated cells (NM) or CSCs (DM) were washed in PBS and 1 × 10^6^ cells were labeled with extracellular anti-CD133 and anti-Lgr5 antibodies as well as a marker of viability (Viobility^TM^ 405/452 Fixable Dye, Miltenyi Biotec, Bergisch Gladbach, Germany). Cells were incubated 30 min at RT in the dark, washed in PBS and then fixed 10 min in 4% paraformaldehyde. After a wash, cells were permeabilized 30 min at 4 °C in Perm buffer III (BD Biosciences, Le Pont de Claix, France). Finally, cells were labeled with intracellular anti-Bmi1, anti-Oct4 and anti-Nanog antibodies for 30 min at RT in the dark. Samples were analyzed with a CytoFLEX flow cytometer (Beckman Coulter, Brea, CA, USA) and Kaluza software (v2.1, Beckman Coulter).

#### 2.5.2. Cell Cycle Analysis

Cells were washed with ice-cold PBS, resuspended in ice-cold 70% ethanol and placed at −20 °C overnight. The next day, after a wash, cells were incubated with RNase A (Sigma Aldrich, St. Louis, MO, USA) and Propidium Iodide (PI) for 20 min before analysis with a CytoFLEX flow cytometer (Beckman Coulter) and ModFit LT™ software (v5.0.9, Verity Software House, Topsham, ME, USA).

### 2.6. Proliferation Assay

Five thousand cells were seeded in a 96-well plate and proliferation rate was measured using BrdU Cell Proliferation Assay Kit (Cell Signaling, Danvers, MA, USA) according to the manufacturer’s instructions. Absorbance was measured at 450 nm with a Multiskan FC Thermo Scientific microplate photometer (Thermo Fisher).

### 2.7. Metabolic Activity Assay

Five thousand cells were seeded in a 96-well plate and treated or not with EVs (from NM or DM cultured cells) for 24 h or 72 h at the same ratio as previously. After EVs treatment, cells were incubated with increasing doses of 5-FU for 48 h (500 µM to 1 µM). Cell toxicity was measured using CellTiter 96^®^ AQueous One Solution Cell Proliferation Assay (Promega, Madison, WI, USA) according to the manufacturer’s instructions and half maximal inhibitory concentration (IC50) was graphically determined (GraphPad Prism 7.04).

### 2.8. Migration and Invasion Assays

Fifty thousand cells were seeded in an Incucyte^®^ Imagelock 96-well plate (Sartorius, Goettingen, Germany) and scratch migration and invasion assays were performed according to the manufacturer’s instructions. Briefly, cells were seeded the day before at around 80% confluency. The next day, wounds were created using the 96-well WoundMaker (Sartorius). After washing the wells twice to remove the floating cells, 100 µL of culture medium (with or without EVs) was added to the wells.

For the invasion assay, after creating the wound, cells were coated with 50 µL of Matrigel^®^ solution (Corning, New York, NY, USA). To polymerize the Matrigel^®^, the plate was incubated for 30 min at 37 °C and then 100 µL of culture medium (with or without EVs) was added to the wells. Wound closure was analyzed every 2 h for 48 h using IncuCyte 2021A software (Sartorius).

### 2.9. Self-Renewal Capacity

Five hundred cells (NM or DM) were seeded in a 96-well plate in defined medium for 7 days and analyzed every 24 h with the Incucyte^®^ system and IncuCyte 2021A software (Sartorius).

### 2.10. Crossover Frequency Measurement

A quadrupole electrode sensor, implemented within a microfluidic chip in which a suspension of individual cells is injected, is used to perform dielectrophoretic characterizations (Figure 1). The setup employs a pressure-driven microfluidic controller which fine-tunes the liquid flow, enabling the accurate, sequential positioning and halting of individual cells over the sensor. We apply a DEP high frequency signal (30 to 500 MHz) to bias the sensor electrodes. At the highest frequency (around 500 MHz), the powerful electric field generated around the cell causes a phenomenon known as DEP trapping, effectively holding the cell in the exact center of the microelectrodes. At such a frequency, the cell experiences a repulsive dielectrophoretic force from the electrodes. This mode of operation is known as negative dielectrophoresis (nDEP). Then, the generator frequency is swept down to screen the intracellular physical properties of each single cell. Cell movement transitions from a “repulsive” state (negative dielectrophoresis or nDEP) in the center of the quadrupole to an “attractive” state (positive dielectrophoresis or pDEP) near the edges of any of the four sensor electrodes, and this transition occurs at a specific frequency of the signal called “crossover frequency (CF)”. These frequency values slightly vary for cell to another, reflecting intracellular biophysical heterogeneity. By analyzing a sufficiently large number of cells, we can then determine a representative dielectrophoretic signature—specifically, the crossover frequency distribution—characteristic of that cell subpopulation. This signature allows for the discrimination of cell subpopulations by measuring the dielectric properties of their cytoplasmic content without any labeling. For more details, please refer to our previous publication [28].

### 2.11. Statistical Analysis

Values are represented by mean ± SEM obtained from at least three independent experiments, except values from DEP analyses, which are represented as mean ± SD. Statistical analysis was performed using PAST software (version 2.17c) to conduct a one-way ANOVA (*: *p* < 0.05, **: *p* < 0.01, ***: *p* < 0.001). Statistical analysis concerning miRNA sequencing data was performed in an R environment (version 4.3.0).

### 2.12. Small RNA Sequencing and Bioinformatics Analysis

Small RNA sequencing was conducted by the Beijing Genomics Institute, following the manufacturer’s protocol for Rolling-Circle and DNA Nano-Ball (DNB^TM^) technology. After sequencing, raw data with adapter sequences or low-quality sequences was filtered. This step was completed by SOAPnuke, which was developed by BGI. Additionally, we performed a second round of quality control using FASTQC (version 0.12.1) and conducted low-quality (Phred quality score < 30) and short-length read filtering (<10 bp) using CutAdapt (version 4.3). The clean reads of each sample were mapped to the human reference genome (GRCh38 assembly) using STAR splice-mapper and read counts for each miRNA were obtained using the feature Counts counting tool and GTF annotation file of miRNA extracted from GRCh38 assembly. Differential miRNA expression was analyzed using DESeq2 to identify differentially expressed miRNA in EV-treated versus untreated SW480 cells according to the following selection criteria: absolute fold change superior to 2 and p-adjusted (after Benjamini–Hochberg (BH) multiple testing correction) inferior to 0.05. At the same time, miRNA-sequencing data obtained from EVs only was processed in the same way to evaluate highly enriched miRNA-containing parts in this subcellular fraction, but no comparison of the differential analysis versus cellular miRNA content was conducted due to a high difference in the size of the libraries.

To obtain biological information about cellular consequences of miRNA deregulation caused by the treatment of SW480 cells with SW620 cell-derived EVs, we looked for predicted or experimentally described mRNA targets of deregulated miRNA using 4 online tools: miRDB (version 6.0), TargetScan (release 8.0), miRTARBase (release 9.0 beta) and DIANNAmicroT (2023 release). After selecting the predicted target from the entire database to obtain high-confidence target genes, we performed a functional enrichment analysis using terms associated with gene ontology (biological process) and biological pathways (KEGG and Reactome) using the Database for Annotation, Visualization, and Integrated Discovery (DAVID) tool.

## 3. Results

### 3.1. Cancer Stem Cells from Colorectal Cancer Cell Lines Exhibit a Lower Crossover Frequency than Their Differentiated Counterparts

**Figure 2:**
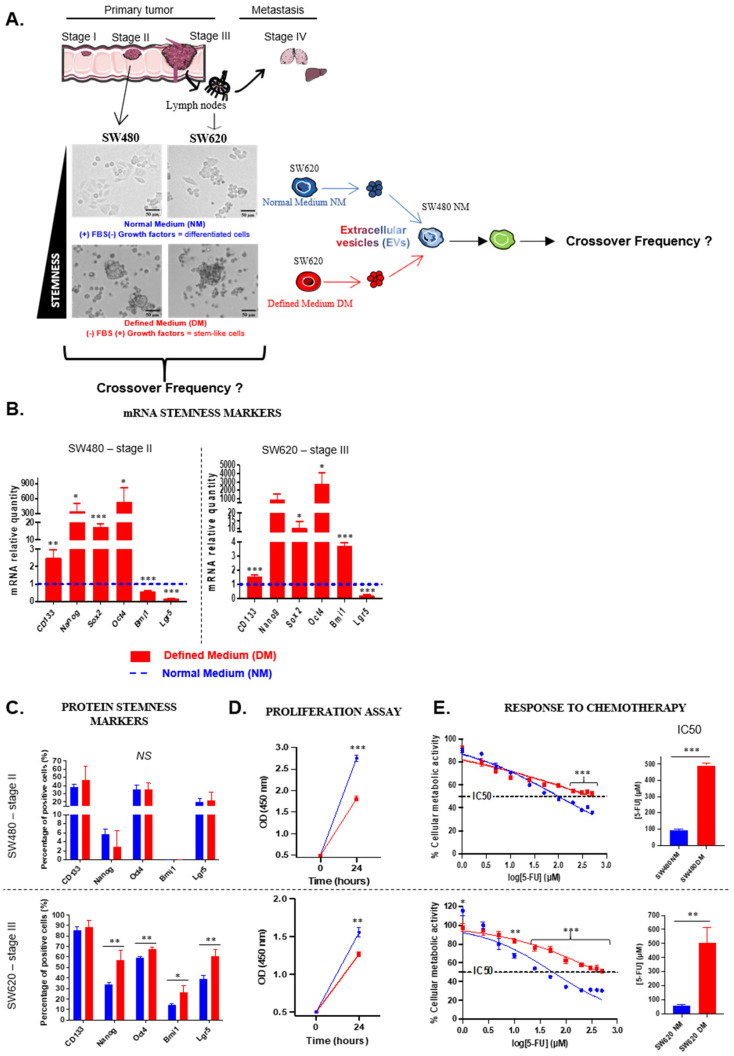

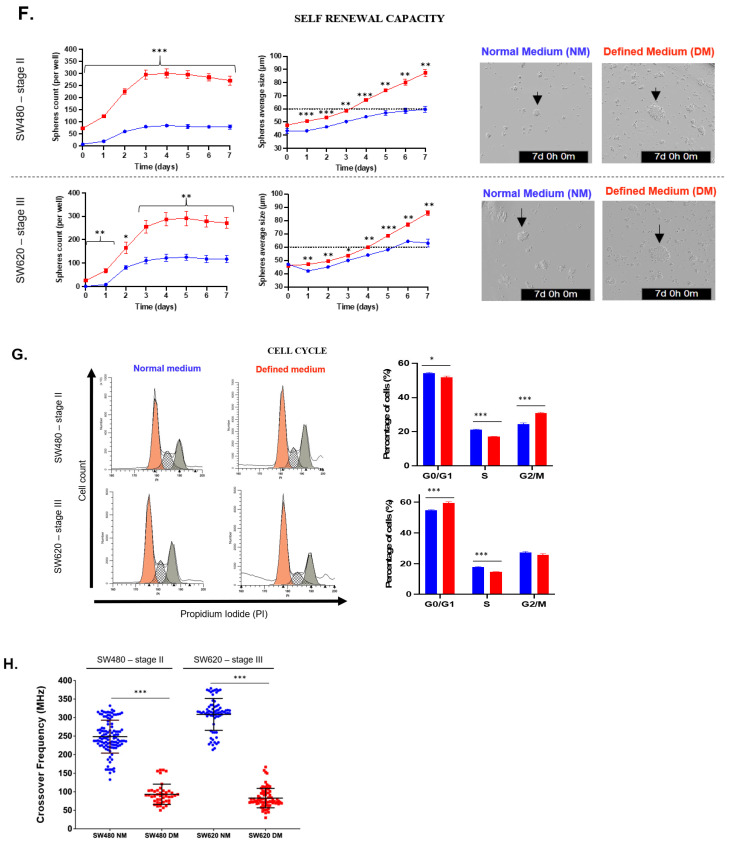
Characterization of CRC cell lines according to culture conditions. Cells were grown under normal (NM) in blue or defined medium (DM) in red to enrich in differentiated cells or CSCs, respectively (**A**). To validate culture conditions, stemness-related genes (**B**) and proteins (**C**) were analyzed. Functional properties were explored with proliferation rate (**D**), response to chemotherapy (**E**), self-renewal capacity (**F**) and cell cycle distribution (**G**). The crossover frequency distribution of both cell types were measured at UHF ((**H**), SW480 NM: 111 cells; SW480 DM: 45 cells; SW620 NM: 73 cells; SW620 DM: 78 cells). All results are represented as mean ± SEM except crossover frequencies, which are represented as mean ± SD, NS *p*-value indicates lack of statistical significance, * *p*-value < 0.05, ** *p*-value < 0.01, *** *p*-value < 0.001 using one-way ANOVA test.

To test the lab-on-a-chip UHF-DEP analysis on CRC, two cell lines derived from the same patient were chosen: SW480 as a stage II and SW620 as a stage III (lymph nodes metastasis). Each cell line was grown under two distinct conditions: in normal medium (NM) to enrich the cell population in differentiated cells or maintained in defined medium (DM) to enrich it in CSCs. Defined medium did not contain FBS but is supplemented with specific growth factors in order to generate cellular stress leading to the acquisition of stem-like properties. As shown in Figure 2A, cells cultured in DM are no longer adherent but grow as colonospheres as a result of the acquisition of “stemness” properties. Enrichment in CSCs was verified by assessing their phenotypic and functional properties. Both cell lines cultured in defined medium overexpressed all stemness-related genes (*CD133*, *Nanog*, *Sox2*, *Oct4*) (Figure 2B). Interestingly, both cell lines underexpressed *Lgr5*, which is a stemness gene related to cell activation, while only SW620-DM cells overexpressed *Bmi1*, which is related to quiescence. The same analysis was performed by flow cytometry to analyze the protein counterparts of those markers (Figure 2C). No significant differences were observed concerning the SW480 cell line and Bmi1 was not even detected regardless of culture conditions. On the other hand, SW620 cells grown under DM overexpressed all stemness markers except CD133 which was already highly expressed by differentiated cells. SW480 cells seemed to require more time in DM than SW620 cells to overexpress these markers, suggesting that stage III-derived cells exhibit a stronger “cancer stem cells” burden. Indeed, regardless of culture conditions, SW620 cells always overexpress stemness-related proteins compared to SW480 cells (Supplementary Figure S1B). Since overexpressing stemness markers is not sufficient to consider cells as CSCs, we explored their functional properties. For both cell lines, cancer stem-like cells presented a lower proliferation rate compared to differentiated cells (Figure 2D). Regarding cell cycle distribution, there was an accumulation of SW480-DM cells in the G2/M phase compared to SW480-NM cells, whereas SW620-DM cells accumulated in the G0/G1 phase compared to their differentiated counterparts (Figure 2G), which is consistent with the aforementioned overexpression of *Bmi1* by those cells. Similarly, cancer stem-like cell enrichment from SW480 cells demonstrated a resistance to chemotherapy by exhibiting a 5-fold higher IC50 than differentiated cells (Figure 2E). Finally, we explored the self-renewal ability of both cell types by growing them in DM for seven days (Figure 2F). For both cell lines, cancer stem-like cells formed more and larger colonospheres than differentiated cells. Altogether, these results allow us to consider cells grown under defined medium as CSCs.

On the other hand, these cell lines can be characterized through their biophysical properties. Indeed, we have previously used “UHF-DEP investigations” (i.e., difference in dielectrophoretic behavior under a non-uniform electric field) on primary cells from brain tumors as well as a glioblastoma cell line to establish their dielectrophoretic signature. Our results demonstrated that CSCs display a lower crossover frequency in an ultra-high frequency range whereas no differences were observed at a low frequency range (where the dielectric properties of the plasma membrane are dominant) [20]. Our previous work highlighted the crossover frequency as a relevant CSC discriminant parameter. Thus, we determined the crossover frequency at UHF regime for our CRC cell lines. We obtained similar results for both CRC cell lines with a lower crossover frequency for CSCs compared to differentiated cells (Figure 2H). Furthermore, we observed a complete change in the distribution of CF between CSCs and differentiated cells. For both cell lines, almost all the differentiated cells exhibited a CF superior to 200 MHz (88% for SW480 and 100% for SW620), while the crossover frequency of CSCs was lower than 200 MHz (Table 1, Supplementary Figure S1).

Although these results confirm that growing cells in DM enriches them in CSCs, the cell lines do not depend on the same phase of the cell cycle. Indeed, SW480-DM cells appear to be a proliferative type of CSCs whereas SW620-DM cells appear to be a quiescent one. This lower crossover frequency of CSCs is consistent with our previous results on glioblastoma and medulloblastoma tumors [28,29].

### 3.2. Extracellular Vesicles Affect the Phenotype and Biophysical Properties of Treated Colorectal Cell Line

**Figure 3:**
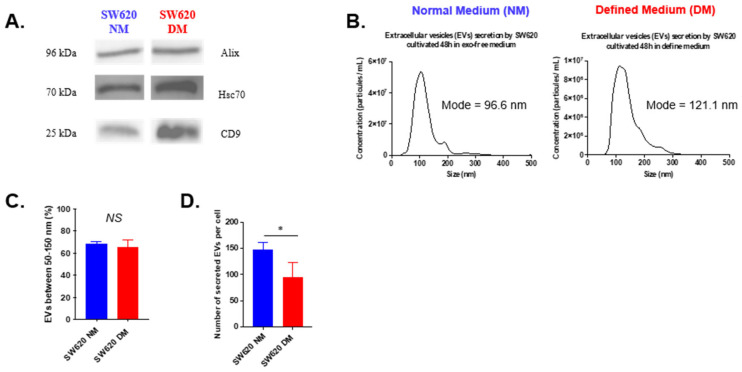
EV characterization. EVs derived from SW620 cells (NM in blue or DM in red) were collected by ultracentrifugation and characterized by Western blot (**A**) and NTA profile (**B**), percentage of EVs (**C**), number of EVs/cells (**D**). All results are represented as mean ± SEM, * *p*-value < 0.05, using one-way ANOVA test.

Since part of an EV’s cargo mirrors that of its parental cells, we hypothesized that CSC-derived EVs might carry protein or nucleic material that could transfer additional aggressive properties to neighboring tumor cells compared to EVs derived from differentiated tumor cells. In this context, we decided to use EVs secreted by the late-stage SW620 cell line to treat the early-stage SW480 cell line to evaluate a putative transfer of aggressiveness. EVs were collected from the supernatant of the SW620 cell line grown in NM or DM conditions (Figure 2A) and their characterization by Western blot and NTA shows that CSCs secrete fewer EVs than differentiated tumor cells (Figure 3A–C).

**Figure 4:**
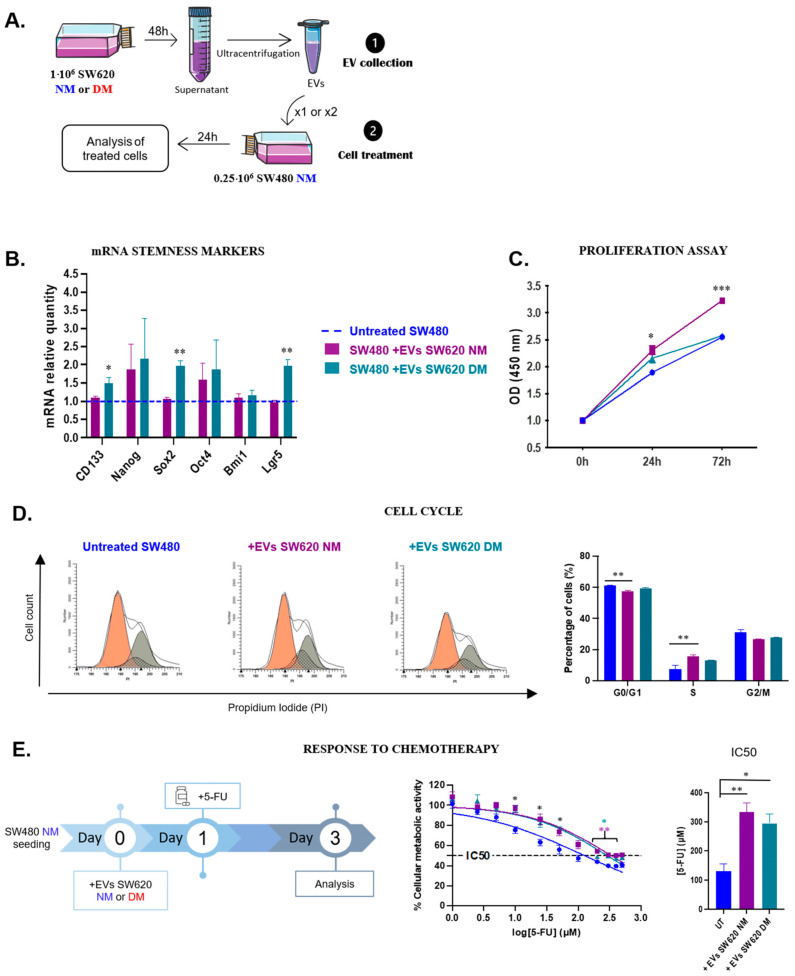

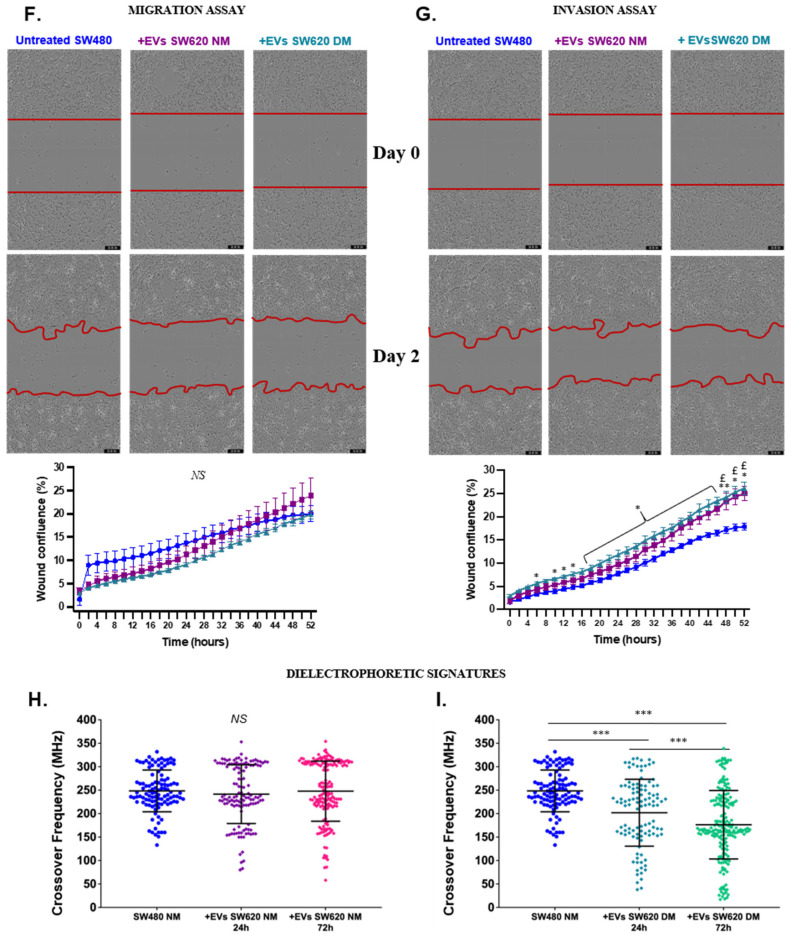
Effects of EVs derived from SW620-CSCs or differentiated cells on the SW480 cell line. To test a transfer of aggressiveness, SW480-NM cells were treated once for 24 h with EVs derived from the SW620 cell line (NM or DM) (**A**). To assess the biological effects of this treatment, stemness-related genes (**B**) were analyzed, as well as the proliferation rate (**C**), cell cycle arrest (**D**) and chemotherapy response (**E**). The ability of treated cells to migrate (**F**) or invade (**G**) was also explored (*: +EVs SW620 versus untreated SW480, £: +EVs SW620 NM versus untreated SW480) and crossover frequencies measured ((**H**,**I**); SW480 NM in blue: 111 cells; SW480 + EVs SW620 NM 24 h in purple: 134 cells; SW480 + EVs SW620 NM 72 h in pink: 171 cells; SW480 + EVs SW620 DM 24 h in cyan: 107 cells; SW480 + EVs SW620 DM 72 h in green: 193 cells). All results are represented as mean ± SEM except crossover frequencies, which are represented as mean ± SD, NS *p*-value indicates lack of statistical significance, * and £ *p*-value < 0.05, ** *p*-value < 0.01, *** *p*-value < 0.001 using one-way ANOVA test.

Those EVs were used to treat SW480-NM cells for 24 h (Figure 4A). When SW480-NM cells were treated with CSC-derived EVs, they overexpressed some stemness transcripts such as *CD133*, *Sox2* and *Lgr5*, whereas no significant differences were observed with EVs derived from differentiated tumor cells (Figure 4B). In contrast, cells treated with EVs derived from differentiated cells demonstrated a higher proliferation rate, increasing overtime, while no differences were observed with CSC-derived EVs (Figure 4C). Strikingly, this lack of effect of CSC-derived EVs suggests that treated cells may be arrested in the G0/G1 phase, as are CSCs. Cell cycle analysis reveals an unchanged distribution after treatment with CSC-derived EVs, whereas EVs derived from differentiated cells induced an accumulation in the S phase (Figure 4D), which is consistent with results obtained previously on proliferation rate. Concerning chemotherapy response, both types of EVs induced a 3-fold increase in the IC50 of treated cells (Figure 4E). Metastasis is a main hallmark of cancer [22], which is why migration and invasion abilities were investigated upon EVs intake. The ability to migrate did not seem to be affected by any kind of EVs (Figure 4F). However, the invasion capacity was enhanced when cells were treated with EVs derived from CSCs or differentiated tumor cells. Nevertheless, CSC-derived EVs affected invasion only 6 h post-treatment whereas the same effect induced by EVs derived from differentiated tumor cells was delayed to 48 h (Figure 4G). These results indicate that EVs directly influence the phenotype of treated cells. Therefore, their crossover frequencies were measured to determine whether their biophysical properties were altered. Cells treated with EVs derived from CSCs exhibit a lower crossover frequency compared to untreated cells (Figure 4I), whereas no significant difference was observed with EVs derived from differentiated cells (Figure 4H). To determine whether this effect was dose-dependent, the cells were treated twice with the same amount of EVs each time for 72 h. Despite a double treatment, crossover frequency from cells treated with EVs derived from differentiated cells was not changed (Figure 4H, pink condition). However, we observe a slight redistribution of CF with the emergence of a few cells with a CF below 100 MHz and 10% more cells exhibiting a CF above 300 MHz when cells were treated once, and 17% when treated twice (Table 2 and Supplementary Figure S2B). Concerning cells treated with CSC-derived EVs, a second treatment led to a greater decrease in crossover frequencies after 72 h (Figure 4I, green condition).

Both kinds of EVs affected the phenotypes of treated cells but in a distinct manner. EVs derived from CSCs triggered the overexpression of some stemness-related genes and those derived from differentiated tumor cells promoted proliferation. In contrast, both types of EVs promoted invasion and metabolic activity. Regarding crossover frequencies, it seems that only CSC-derived EVs affected the biophysical properties of treated cells. Based on these results, we can hypothesize that crossover frequency and biological changes induced by CSC-derived EVs might be closely linked. That suggests this signature might be a useful tool to monitor the acquisition of stemness properties and abilities by tumor differentiated cells.

### 3.3. Cancer Stem Cells from Patient-Derived Primary Cells Exhibit a Lower Crossover Frequency than Their Differentiated Counterparts

**Figure 5:**
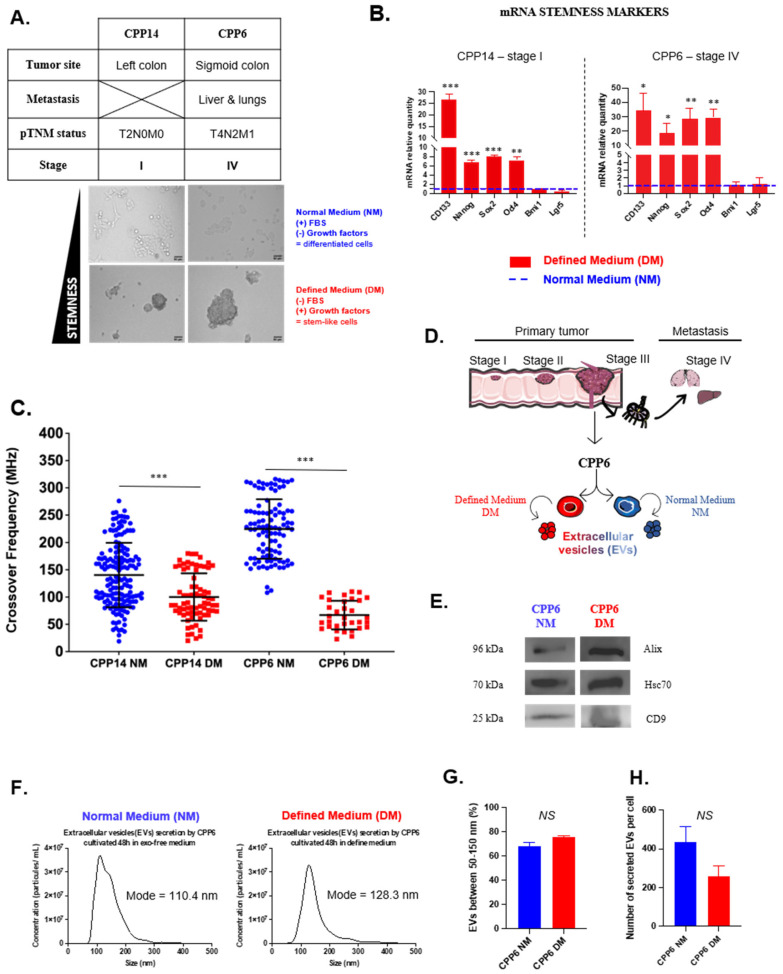
Characterization of patient-derived primary cells and their EVs depending on culture conditions. Cells were grown under NM in blue or DM in red to enrich in differentiated cells or CSCs (**A**). To validate culture conditions, we measured the expression of stemness-related genes (**B**) and crossover frequencies ((**C**), CPP14 NM: 160 cells; CPP14 DM: 73 cells; CPP6 NM: 100 cells; CPP6 DM: 33 cells). CPP6-derived EVs (NM or DM) were collected by ultracentrifugation (**D**) and characterized by Western blot (**E**) and NTA (**F**–**H**). All results are represented as mean ± SEM except crossover frequencies, which are represented as mean ± SD, NS *p*-value indicates lack of statistical significance, * *p*-value < 0.05, ** *p*-value < 0.01, *** *p*-value < 0.001 using one-way ANOVA test.

To achieve a proof of concept in clinical conditions, the same experiments were performed on two CRC patient-derived primary cells (CPP): CPP14 from a stage I and CPP6 from a stage IV of tumor patient. As previously, cells were grown in normal or defined medium. Under the DM condition, they formed colonospheres (Figure 5A) and upregulated most stemness-related genes (Figure 5B). As for cell lines, crossover frequency was weaker in CSC than those observed in differentiated tumor cells (Figure 5C) even though a big overlap was observed for CPP14 (72% of differentiated cells under 200 MHz against 100% of CSCs). Regarding CPP6, the CFs of all differentiated tumor cells were between 100 and 400 MHz while only 15% of cells had CF values related to those of the CSCs (Table 3 and Figure S3B).

As for the cell lines, we observed the same pattern of a distinct lower crossover frequency for CSCs compared to differentiated tumor cells.

### 3.4. Extracellular Vesicles Affect the Phenotype and Biophysical Properties of Treated Patient-Derived Primary Cells

**Figure 6:**
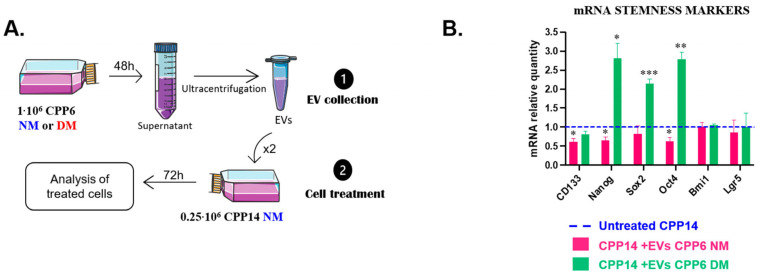

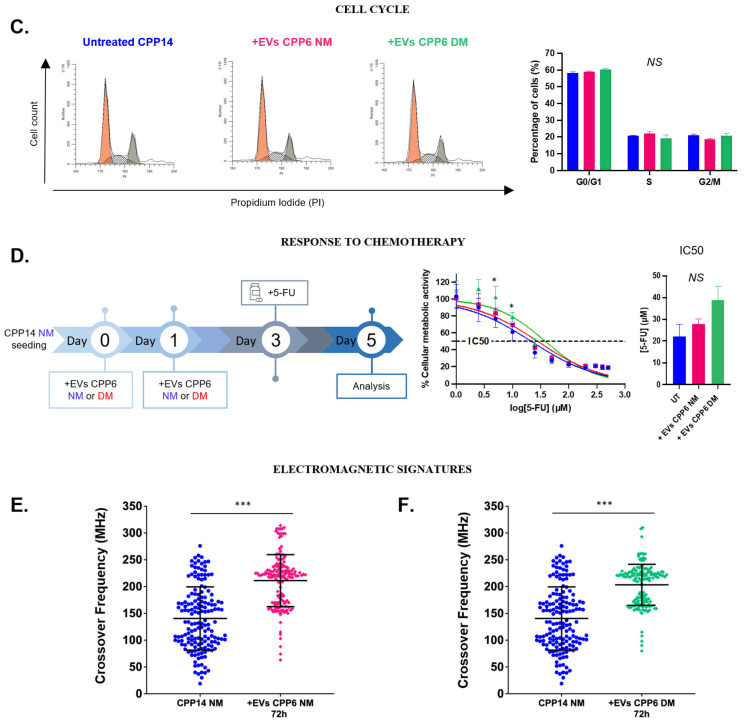
Effects of EVs derived from CPP6-CSCs or differentiated cells on CPP4 primary cells. EVs derived from CPP6 cells (NM or DM) were used to treat CPP14-NM cells (**A**). To assess the biological and biophysical effects of this treatment, expression of stemness-related genes (**B**), cell cycle arrest (**C**), response to chemotherapy (Untreated CPP14 in blue; CPP14 + EVs CPP6 NM in pink; CPP14 + EVs CPP6 DM in green) (**D**) and crossover frequency distribution chemotherapy (Untreated CPP14 in blue; CPP14 + EVs CPP6 NM in pink; CPP14 + EVs CPP6 DM in green) ((**E**,**F**), CPP14 NM in blue: 160 cells; CPP14 NM + EVs CPP6 NM 72 h in pink: 162 cells; CPP14 NM + EVs CPP6 DM 72 h in green: 142 cells) were analyzed. All results are represented as mean ± SEM except crossover frequencies, which were represented as mean ± SD, NS *p*-value indicates lack of statistical significance, * *p*-value < 0.05, ** *p*-value < 0.01, *** *p*-value < 0.001 using one-way ANOVA test.

To demonstrate a transfer of aggressiveness mediated by EVs, CPP14-differentiated tumor cells were treated with CPP6-derived EVs. As previously, CPP6 cells were cultured under normal or defined medium and EVs collected by ultracentrifugation (Figure 5D). Their characterization by NTA demonstrates that CSCs tend to secrete fewer EVs than differentiated tumor cells (Figure 5H). We previously observed stronger effects when cell lines were treated twice with EVs. So, we decided to keep this condition only (Figure 6A). CSC-derived EVs induced an upregulation of *Nanog*, *Sox2* and *Oct4* whereas EVs derived from differentiated tumor cells induced a downregulation of *CD133*, *Nanog* and *Oct4* in treated cells (Figure 6B). In contrast, no significant differences were observed regarding cell cycle arrest (Figure 6C) or response to chemotherapy, although it seems that CSC-derived EVs increased the metabolic activity of treated cells (Figure 6D). Strikingly, we observed an increase in crossover frequencies regardless of EV type, suggesting that the expression pattern of stemness-related genes was not directly linked to the cells’ biophysical properties in primary cell cultures (Figure 6E,F, Table 4).

Both types of EVs alter treated cells leading to a higher crossover frequency. Biologically, we only observe an overexpression of some stemness-related genes when cells are treated with CSC-derived EVs, as well as a trend towards an increased metabolic activity.

### 3.5. Extracellular Vesicles Derived from CSCs Affect the Phenotype and Biophysical Properties of Treated Primary Cells Derived from the Same Patient

**Figure 7:**
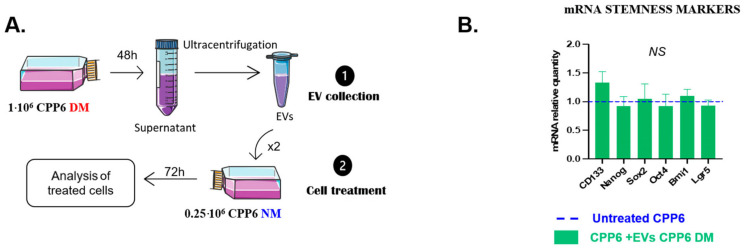

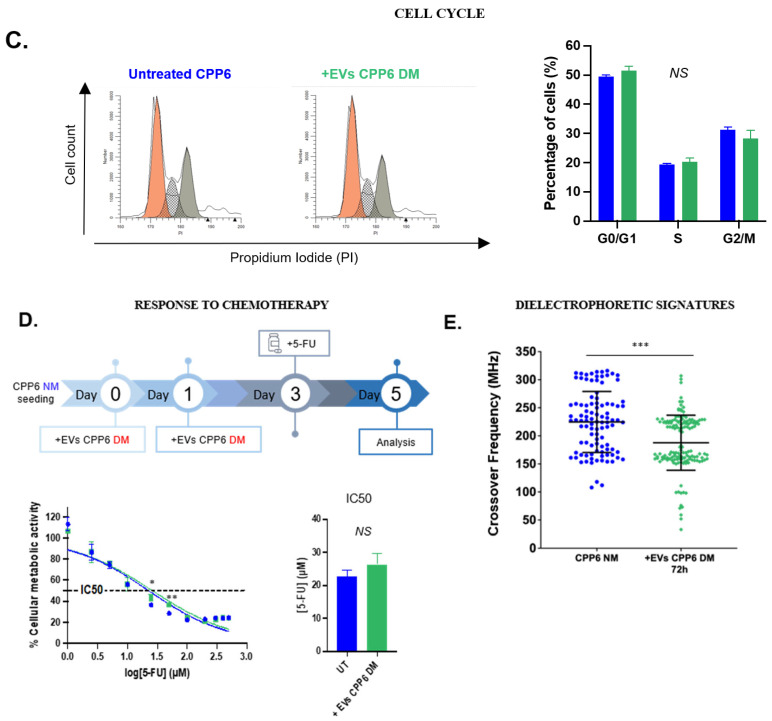
Effects of EVs derived from CSCs on CPP6 primary cells. EVs from CPP6 grown in defined medium were used to treat CPP6-NM cells twice for 72 h. Untreated CPP6 NM are in blue and CPP6 NM treated with EVs from CPP6 DM are in green (**A**). To evaluate the biological effects of such treatment, stemness-related genes (**B**), cell cycle arrest (**C**), response to chemotherapy (**D**) and crossover frequencies (**E**), CPP6 NM: 102 cells; CPP6 NM + EVs CPP6 DM 72 h: 142 cells) were analyzed. All results are represented as mean ± SEM except crossover frequencies, which are represented as mean ± SD, NS *p*-value indicates lack of statistical significance, * *p*-value < 0.05, ** *p*-value < 0.01, *** *p*-value < 0.001 using one-way ANOVA test.

CPP14 and CPP6 were derived from two patients with distinct tumor stages, thus they grew in different tumor microenvironments and have distinct genetic profiles. We wondered what would happen if we used the same primary cells to test a transfer of aggressiveness. To test this hypothesis, differentiated cells were treated with EVs derived from CSCs (Figure 7A). Unfortunately, no significant difference was observed regarding the expression of stemness markers, cell cycle distribution or response to 5-FU (Figure 7B–D). Nevertheless, we observed a lower crossover frequency, suggesting a modification of the biophysical properties of treated cells (Figure 7E).

No biological effects were shown when CPP6-NM cells were treated with EVs derived from CSCs although their crossover frequency was changed. This observation does not imply that nothing occurred at the biological level, suggesting that the properties we analyzed were not modified by EV intake. While conventional biological assays provide highly specific molecular insights—often targeting the expression of a single protein—UHF-DEP analysis offers a broader, integrative view of the cell by capturing its global biophysical properties and overall phenotypic profile. Global intern modifications, such as cytoplasm composition or volume ratio between nucleus and cytoplasm, often lead to a changed crossover frequency. Moreover, this result highlights the relevance of the crossover frequency in detecting phenotypic transformation of cells and its complementarity with conventional biological analyses in revealing modifications that may go unnoticed when appropriate molecular assays are not employed. (Table 5).

### 3.6. Extracellular Vesicles Derived from CSCs Induce a Deregulation of miRNAs in Treated Cells

**Figure 8:**
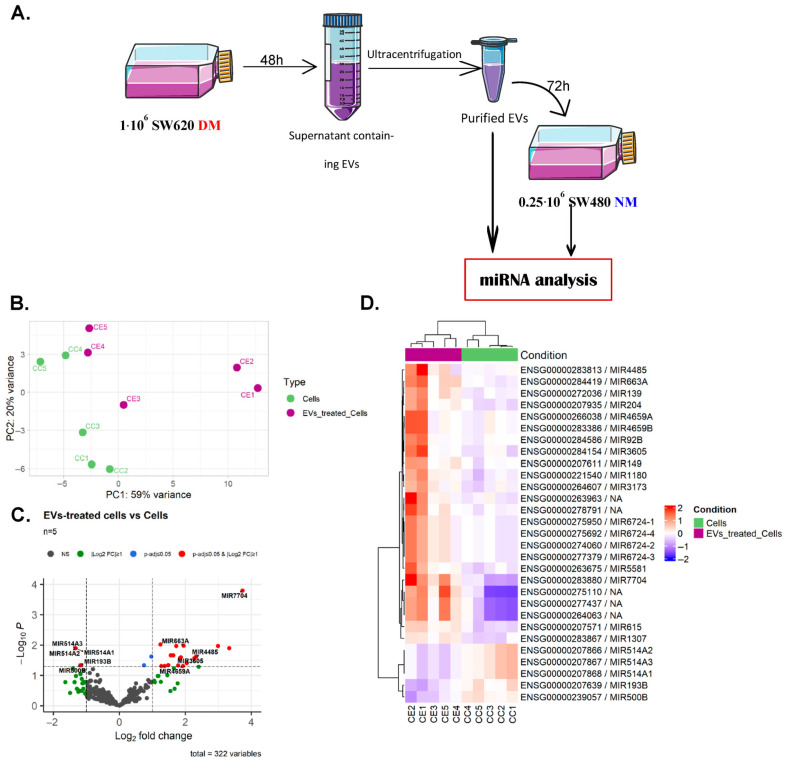
miRNA profiling of SW480 cells treated with CSC-derived EVs. Workflow before miRNA analysis (**A**). For other panels: PCA of miR-Seq data obtained from SW480 cells treated or not treated with EVs, performed on regularized log (rlog) transformation count data after normalization and filtration of low and constant expressed miRNAs (DESeq2 and HTS Filter R packages 4.3.0). Five independent replicates were performed for each condition: untreated control cells (noted CC, green symbols) and EV-treated cells (noted CE, purple symbols) (**B**). Volcano plot showing miRNAs differentially expressed according to selection threshold, i.e., adjusted *p*-value ≤ 0.05 and log2 fold change ≥ 1 induced by treatment of cells with EVs: 24 miRNAs are upregulated and 5 are downregulated after treatment. Gene symbols correspond to the top 5 up- and downregulated miRNAs (**C**). Heatmap of the 29 miRNAs differentially expressed in EV-treated cells compared to untreated cells. Gene expression intensities were rlog-transformed and centered on the median. They are represented by a color gradient from blue to red, reflecting, respectively, low and high levels of gene expression in the cells. miRNAs (rows) and samples (columns) are clustered hierarchically using Pearson’s correlation distance and the average linkage method (**D**).

MicroRNAs (miRNAs) are known to be part of EVs’ cargo and have been described to affect numerous cellular modifications as well as tumor progression processes, including the acquisition of an aggressive phenotype. We hypothesized that crossover frequency modification could be due to miRNA transfer carried by EVs. Both the SW480 and SW620 cell lines came from the same patient but at different stages of the disease. In order to understand how SW480 cells become more aggressive (leading to SW620 cells), the miRNA content of the EVs was first analyzed, then SW480 cells were treated with EVs derived from SW620 cells grown in a defined medium, and the change in miRNA expression was analyzed (Figure 8A).

Among the 96 most abundant miRNAs detected in CSC-derived EVs (miRNAs with counts superior to Q3 value of read count distribution), 95 were found in the VesiclePedia or Exocarta databases (77 in both). The two most abundant ones are miR-21 and miR-27B, which are well-known to be overexpressed in CRC and associated with drug resistance. To estimate the biological impact of EVs on treated cells, we focused on the 25 most abundant miRNAs detected in EVs and searched for high-confidence mRNA targets, predicted by four predicting tools. We performed functional enrichment analysis on 826 common mRNA targets on biological process terms (GO, KEGG and Reactome) and retrieved a high proportion of enriched signaling pathways related to cancer stem cell biology (such as “Signaling pathways regulating pluripotency of stem cells” or “FOXO-mediated transcription”) (Figure S5).

In parallel, miRNA profiling was conducted on SW480 cells treated with CSC-derived EVs versus untreated cells. Principal component analysis (PCA) performed on miRNA expression profiles shows the expected separation between untreated and treated cells, even though biological replicates are dispersed (Figure 8B). Differential analysis highlights 24 upregulated and 5 downregulated miRNAs in cells treated with CSC-derived EVs, with a log_2_ fold change ranging from 3.7 to −1.3 (Figure 8C, Table S1). Clustering analysis confirms the clear separation of two cell types based on the 29 differentially expressed miRNAs (Figure 8D).

The same in silico strategy was applied to confirm the potential molecular changes induced on treated cells, extrapolated from the miRNA content of CSC-derived EVs: we searched for high-confidence mRNA targets predicted by four predicting tools for the 24 upregulated miRNAs in treated cells. Functional enrichment analysis of 625 common mRNA targets confirmed the enrichment of signaling pathways related to cancer stem cell biology (such as “PI3K-Akt signaling pathway” or “Cellular Senescence”) (Figure S5).

Comparing the two lists of mRNA targets predicted with high confidence for the most abundant miRNAs in EVs and the most commonly deregulated miRNAs in treated cells (826 and 625 target mRNAs, respectively), we found that 129 genes were common. These mRNAs were involved in numerous signaling pathways (MAPK, EGFR tyrosine kinase inhibitor, PIK3-Akt, TGFβ, etc.), suggesting molecular reprogramming, most likely inducing a potential change in cell content (Figure S6). This is in line with the previous results obtained using our biophysical approach (Figure 4).

## 4. Discussion

This study highlights the relevance of our lab-on-a-chip sensor which can perform UHF-DEP analysis to discriminate subpopulation of cancer cells and track their phenotypic transformation. Previously, this method allowed us to distinguish CSCs from differentiated tumor cells in two types of brain tumor. In both cases, the study revealed that CSCs exhibit a distinct and consistently lower crossover frequency than differentiated tumor cells [28,29]. The first aim of this work was to apply this technology to CRC to determine whether the observations made in brain tumors could also be achieved in other tumor types. The second aim was to demonstrate that crossover frequencies could follow cell changes when they were treated with EVs derived from aggressive cells such as CSCs. By growing cells in a defined medium to enrich them in CSCs, we observed that CSCs exhibit a lower crossover frequency than differentiated tumor cells for both cell lines and patient-derived primary cells.

The characterization and discrimination of CSCs in a heterogeneous tumor mass remain a challenge since their discovery two decades ago. The scientific consensus initially held that the presence of stemness markers was adequate to identify CSCs, and for many years much effort was expended to find the most relevant markers or characterization techniques to identify such cells [5]. As these markers are also expressed by normal stem cells, only their level of expression could distinguish normal from cancer stem cells. Functional properties were then assessed to complement phenotypic characterization and overcome the lack of universal CSC markers. The notion of CSC plasticity has made it even more difficult to characterize and detect cancer stem cells [22]. Because of this dynamic plasticity, it is more complicated to qualify a cell as a CSC on the basis of phenotypic and functional characterizations. Moreover, these techniques are expensive and time-consuming and require special equipment that is not always available in research laboratories. In contrast, UHF-DEP analysis allows us to characterize and discriminate CSCs with a label-free method in a very short period of time (about an hour).

In recent years, EVs have drawn growing interest due to their ability to transfer aggressive properties to cells in the tumor microenvironment [2,11,12,14,15,16,30]; we wanted to test whether the lab-on-a-chip UHF-DEP sensor could detect a phenotypic change induced by EV intake. We started our investigations with two cell lines derived from the same CRC patient and treated the early-stage cell line (SW480-NM) with EVs derived from the late-stage cell line (SW620-NM or SW620-DM). When differentiated SW480 cells were treated with CSC-derived EVs (SW620-DM), there was an overexpression of some stemness-related genes (*CD133*, *Sox2*, *Lgr5*). In contrast, treating those cells with EVs derived from differentiated cells (SW620-NM) increased their proliferation rate. Finally, their ability to resist chemotherapy increased threefold after being treated with both types of EVs. No change in crossover frequency was detected when cells were treated with EVs derived from differentiated cells (compared to untreated cells), suggesting that their cargo is unlikely to increase in aggressiveness and/or induce the acquisition of stemness properties. On the other hand, when SW480-NM cells were treated with CSC-derived EVs (SW620-DM), their crossover frequency was significantly decreased compared to untreated cells. Similar experiments were also performed on primary CRC cells derived from two patients. Treatment of early-stage primary cells (CPP14) with EVs derived from late-stage primary cells (CPP6) was performed to determine if EVs were able to transfer aggressiveness as previously demonstrated in glioblastoma [11]. As expected, gene expression analysis confirmed that only CPP14 treated with CSC-derived EVs (CPP6-DM) overexpressed genes related to stemness (*Nanog*, *Sox2*, *Oct4*), suggesting that the ability of EVs to transfer aggressiveness depends on the cell of origin. Our results also show that cell cycle distribution is not altered by EV intake and that metabolic activity tends to increase with the internalization of CSC-derived EVs. Strikingly, crossover frequencies increased regardless of EV types, indicating a phenotypic change even for cells treated with EVs derived from differentiated cells, while no biological changes were detected. This result suggests that this lab on a chip could help detect modifications that might be difficult to find evidence of with conventional biological approaches. In contrast, our latest experiment, in which we used EVs derived from CPP6-DM cells to treat CPP6-NM cells, revealed that they retained similar phenotypic characteristics despite EV intake. However, their crossover frequency was significantly reduced after treatment, confirming that analysis of their biophysical properties is relevant for detecting modifications related to cellular plasticity that can potentially go unnoticed in biology [31]. This UHF-DEP sensor provides relevant results without prior labeling at a very moderate cost. It can be used to distinguish CSCs from differentiated cells in several types of solid tumors and also to monitor the acquisition and evolution of aggressive characteristics of cells [28,29,32,33]. We used it to track alterations induced by EV intake, but it could also be useful to detect EVs in liquid biopsies and monitor their aggressive potential even before their content is analyzed.

There are other approaches using DEP to characterize EVs, but most of these techniques are directly applied to EVs and do not analyze the impact of EVs and the transfer of their contents to target cells [34]. Analysis of how the crossover frequency of EV impacts targeted cells would complement their biological analysis and enable assessment of tumor heterogeneity and the risk of relapse induced by the transfer of genetic and protein material from therapy-resistant cells [35]. In addition, the development of a cell-sorting device based on UHF-DEP technology could be used to isolate CSCs from the tumor mass. The isolation of CSCs will be faster and less expensive than in conventional techniques based on their phenotypic and functional properties, without altering such properties through labeling. Since it is known that a high number of cancer stem cells correlates with a high risk of relapse in CRC [4,7], this tool would allow for potential early detection of the CSC subpopulation in the tumor and thus tailor treatment of CRC patients.

It is now well established that EVs contain a variety of molecular constituents, including miRNAs or small non-coding RNAs, which play an important role in post-transcriptional regulation of gene expression. According to the Exocarta exosome database (http://exocarta.org), 1638 miRNAs have been identified in Homo Sapiens EVs. Among the 96 most abundant miRNAs detected in SW620-CSC-derived EVs, 92 were found in Exocarta, confirming their origin. Among them, miR-21 is the most abundant and, interestingly, has already been shown to be significantly upregulated in EVs of colon cancer cells compared to normal human colon epithelial cells and to induce 5-FU resistance by targeting mRNA in treated cells [35]. Moreover, transfer of miR-21 by EVs derived from stromal cells confers paclitaxel resistance to ovarian tumor cells [15,36]. The second most abundant is miR-27b, which may act as a tumor suppressor or oncogene [37]. In silico prediction of the impact of CSC-derived EVs on treated cells demonstrates that many signaling pathways, often associated with stemness, can potentially be altered. Similarly, this potential is also demonstrated in reverse by an in silico analysis of the intracellular changes that can be predicted by the upregulation of the 24 miRNAs highlighted in SW480 cells treated with EVs. These bioinformatic predictions of changes in molecular profiles in recipient cells must be confirmed by experimental approaches. MicroRNA involvement in cancer aggressiveness and resistance to therapy is now well established as well as the fact that EVs are great providers of miRNAs. However, analysis of miRNA expression profiles in cells is not commonly used in routine clinical practice. Indeed, current methods to extract and analyze miRNAs remain expensive and time-consuming. The use of crossover frequencies could be a faster alternative for identifying miRNA-induced cellular changes and thus monitoring cancer patients during treatments. In this context, crossover frequencies could serve as tools for predicting the risk of recurrence. This new technology could improve patient monitoring and assist with developing tailored therapeutic strategies.

For the first time, our results demonstrated the extreme sensitivity of the biosensor. We wondered about the elements provided by EVs that were likely to change the crossover frequency of the cells. The most obvious candidates are miRNAs. They are widely described as impacting the phenotype of the cells that integrate them. The next steps for biosensor technology will involve developing a DEP cytometer tool for sorting cell populations with the same crossover frequencies. This will enable highly homogeneous populations of cells with the same “electromagnetic signature” to be characterized from a biological point of view. This approach will make it possible to identify links between different crossover frequencies observed in different cancer cells and specific functional or phenotypic characteristics. This could enable the creation of tools for characterizing cells without prior labeling.

## Figures and Tables

**Table 1:** CF distribution under UHF regime for CRC cell lines.

	SW480		SW620	
	Normal Medium (NM)	Defined Medium (DM)	Normal Medium (NM)	Defined Medium (DM)
0–100 MHz	0%	70%	0%	78%
100–200 MHz	12%	30%	0%	22%
200–300 MHz	68%	0%	24%	0%
300–400 MHz	20%	0%	76%	0%

**Table 2:** CF distribution under UHF regime for SW480 cells treated with EVs.

	SW480 NM	+EVs SW620 NM		+EVs SW620 DM	
	Untreated Cells	24 h	72 h	24 h	72 h
0–100 MHz	0%	3%	2%	12%	15%
100–200 MHz	12%	19%	19%	31%	51%
200–300 MHz	68%	48%	42%	45%	27%
300–400 MHz	20%	30%	37%	11%	8%

**Table 3:** CF distribution under UHF regime for patient-derived primary cells.

	CPP14		CPP6	
	Normal Medium (NM)	Defined Medium (DM)	Normal Medium (NM)	Defined Medium (DM)
0–100 MHz	29%	55%	0%	85%
100–200 MHz	53%	45%	35%	15%
200–300 MHz	18%	0%	49%	0%
300–400 MHz	0%	0%	16%	0%

**Table 4:** CF distribution under UHF regime for CPP14 treated with EVs.

	CPP14	+EVs CPP6 NM	+EVs CPP6 DM
	Normal Medium (NM)	72 h	72 h
0–100 MHz	0%	2%	2%
100–200 MHz	12%	34%	37%
200–300 MHz	68%	60%	60%
300–400 MHz	20%	5%	1%

**Table 5:** CF distribution under UHF regime for CPP6 treated with CSC-derived EVs.

	CPP6	+EVs CPP6 DM
	Normal Medium (NM)	72 h
0–100 MHz	0%	7%
100–200 MHz	35%	46%
200–300 MHz	49%	45%
300–400 MHz	16%	2%

## Data Availability

The original contributions presented in this study are included in the article/Supplementary Material. Further inquiries can be directed to the corresponding author.

## Supplementary Materials

The following supporting information can be downloaded at: https://www.mdpi.com/article/10.3390/bios16010002/s1, Table S1: List of 29 miRNAs differentially expressed in cells treated with CSC-derived EVs compared to untreated cells; Figure S1: Characterization of cell lines according to culture conditions; Figure S2: Effects of EVs derived from SW620-CSCs or differentiated cells on the SW480 cell line; Figure S3: Characterization of patient-derived primary cells according to culture conditions; Figure S4: Effects of EVs derived from CPP6-CSCs or differentiated cells on the crossover frequencies of primary cells; Figure S5: In silico prediction of the biological effect of SW480 cells treatment with CSC-derived EVs; Figure S6: Functional Enrichment Analysis of genes commonly targeted by miRNAs detected in CSC-derived EVs or deregulated in treated SW480 cells.
