# Supplementary material for: Ultra-High-Frequency-Dielectrophoresis Microfluidic Biosensor to Detect the Transformation Potential of Extracellular Vesicles Derived from Cancer Stem Cells
Source: Biosensors (Basel). 2025 Dec 19;16(1):2. doi: 10.3390/bios16010002 (PMC12838799; doi:10.3390/bios16010002)
Supplement: Supplementary file 1 [file biosensors-16-00002-s001.zip › biosensors-3937122-supplementary.pdf]

Supplementary Materials

Ensembl ID	log2 Fold Change	adjusted p-value	Gene Symbol	Gene Name	Gene ID
ENSG00000283880	3,73	1,6E-04	MIR7704	microRNA 7704	102465802
ENSG00000275110	3,33	1,3E-02	NA	NA	NA
ENSG00000277437	2,99	1,1E-02	NA	NA	NA
ENSG00000264063	2,99	1,1E-02	NA	NA	NA
ENSG00000263963	2,33	2,4E-02	NA	NA	NA
ENSG00000283813	2,27	2,7E-02	MIR4485	microRNA 4485	100616263
ENSG00000284154	2,04	3,9E-02	MIR3605	microRNA 3605	100500853
ENSG00000284419	1,95	1,0E-02	MIR663A	microRNA 663a	724033
ENSG00000266038	1,93	4,9E-02	MIR4659A	microRNA 4659a	100616348
ENSG00000283386	1,93	4,9E-02	MIR4659B	microRNA 4659b	100616372
ENSG00000278791	1,87	2,5E-02	NA	NA	NA
ENSG00000263675	1,84	2,7E-02	MIR5581	microRNA 5581	100847010
ENSG00000284586	1,78	4,7E-02	MIR92B	microRNA 92b	693235
ENSG00000272036	1,72	1,1E-02	MIR139	microRNA 139	406931
ENSG00000207935	1,63	2,2E-02	MIR204	microRNA 204	406987
ENSG00000221540	1,56	2,2E-02	MIR1180	microRNA 1180	100302256
ENSG00000274060	1,48	4,6E-02	MIR6724-2	microRNA 6724-2	103504727
ENSG00000277379	1,48	4,6E-02	MIR6724-3	microRNA 6724-3	103504739
ENSG00000275692	1,48	4,6E-02	MIR6724-4	microRNA 6724-4	103504733
ENSG00000275950	1,48	4,6E-02	MIR6724-1	microRNA 6724-1	102465433
ENSG00000264607	1,37	4,9E-02	MIR3173	microRNA 3173	100422981
ENSG00000207611	1,27	4,9E-02	MIR149	microRNA 149	406941
ENSG00000207571	1,25	9,6E-03	MIR615	microRNA 615	693200
ENSG00000283867	0,97	2,4E-02	MIR1307	microRNA 1307	100302174
ENSG00000207639	**-1,16**	4,6E-02	MIR193B	microRNA 193b	574455
ENSG00000239057	**-1,18**	4,6E-02	MIR500B	microRNA 500b	100422911
ENSG00000207868	**-1,32**	1,3E-02	MIR514A1	microRNA 514a-1	574516
ENSG00000207866	**-1,33**	1,3E-02	MIR514A2	microRNA 514a-2	574517
ENSG00000207867	**-1,33**	1,3E-02	MIR514A3	microRNA 514a-3	574518

Table S1. List of 29 miRNAs differentially expressed in cells treated with CSCs-derived EVs compared to untreated cells, according to selection threshold (log2 fold change ≥ 1 and adjusted p-value by Benjamini-Hochberg correction ≤ 0.05). miRNAs also found in EVs alone (from SW620-DM cells) are highlighted in grey.

PROTEIN STEMNESS MARKERS

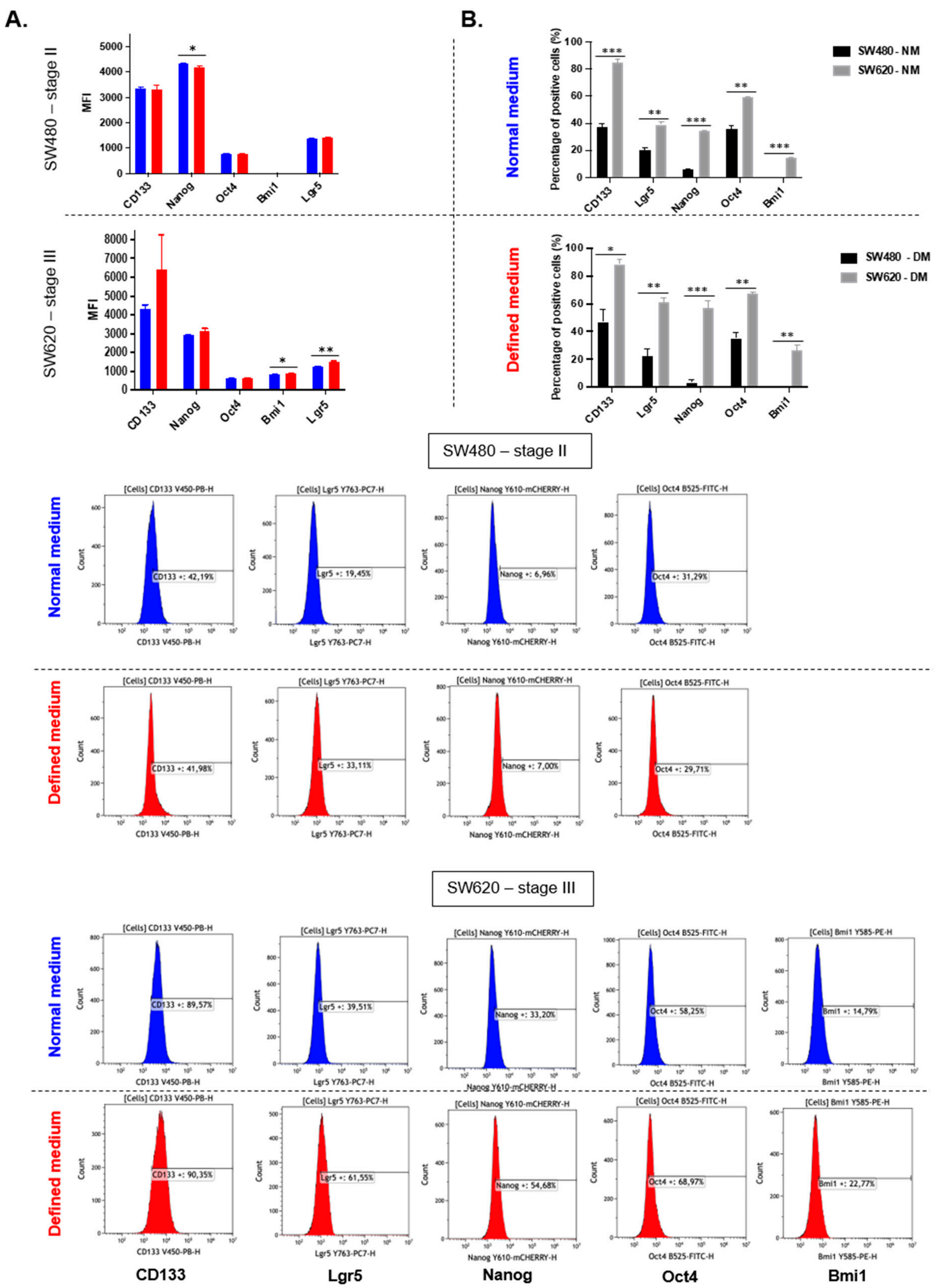

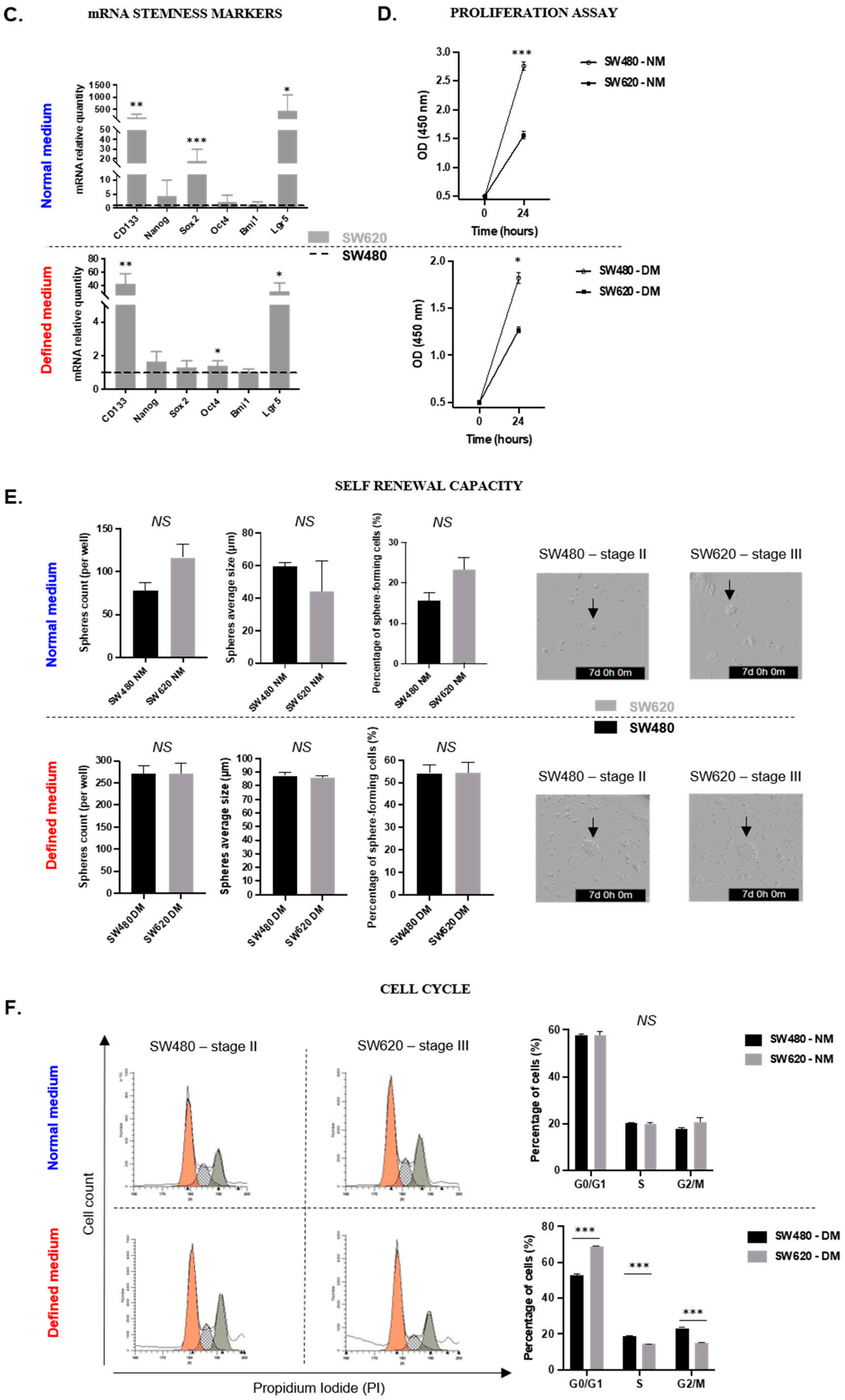
C.
mRNA STEMNESS MARKERS
Normal medium
mRNA relative quantity
CD133
Nanog
Sox2
Oct4
Bmi1
Lgr5
SW620
SW480
Defined medium
D.
PROLIFERATION ASSAY
SW480 - NM
SW620 - NM
OD (450 nm)
Time (hours)
SW480 - DM
SW620 - DM
E.
SELF RENEWAL CAPACITY
NS
Spheres count (per well)
Spheres average size (µm)
Percentage of sphere-forming cells (%)
SW480 NM
SW620 NM
SW480 DM
SW620 DM
SW480 – stage II
SW620 – stage III
7d 0h 0m
F.
CELL CYCLE
Cell count
Propidium Iodide (PI)
Percentage of cells (%)
G0/G1
S
G2/M

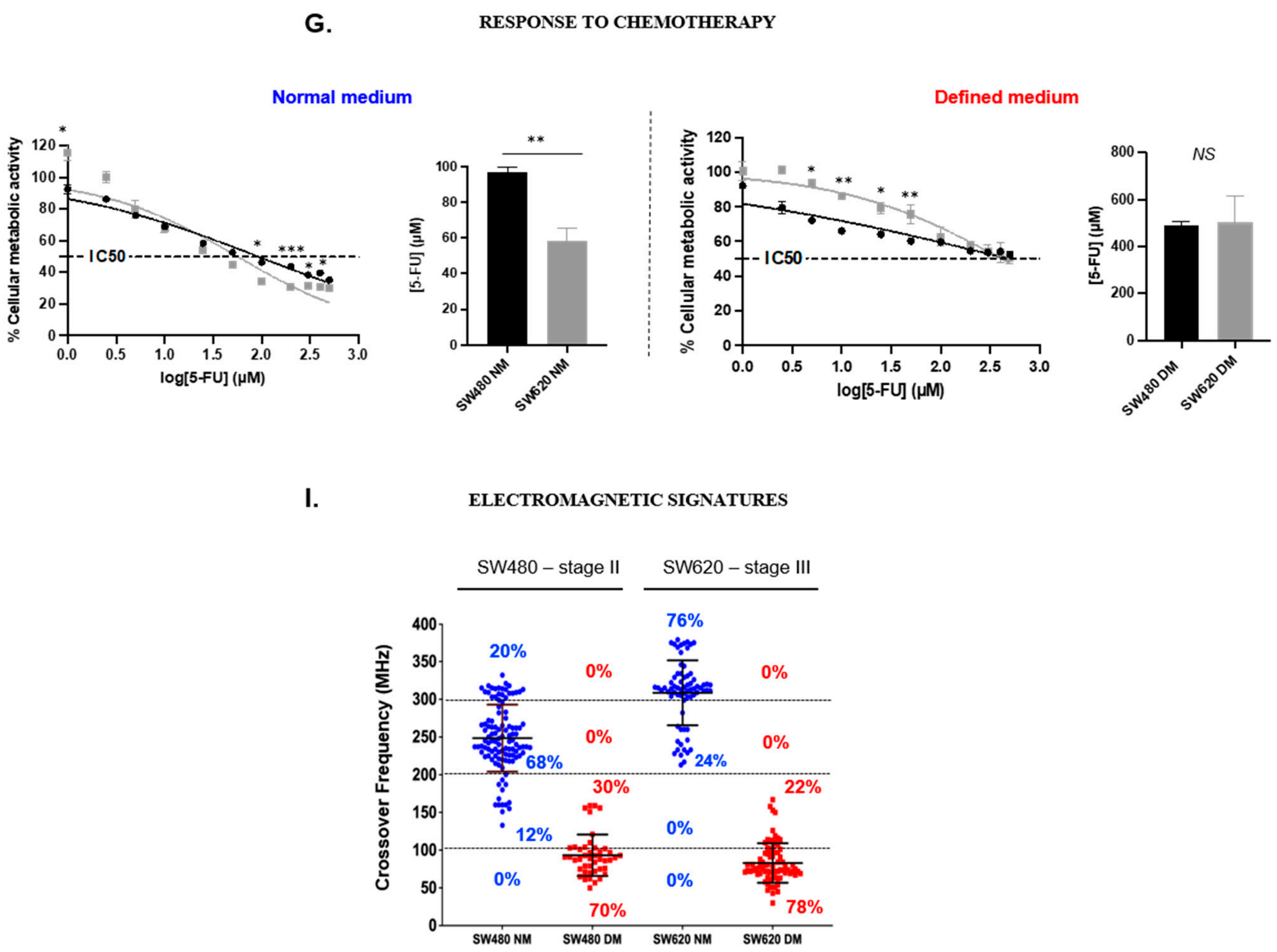

Figure S1. Characterization of cell lines according to culture conditions. Two CRC cell lines (SW480 and SW620) were grown in normal (NM) or defined (DM) medium to enrich in differentiated cells or CSCs, respectively. Cell lines were compared to each other according to culture conditions regarding the expression of stemness markers (A-C), proliferation rate (D), self-renewal capacity (E), cell cycle distribution (F) and response to chemotherapy (G). The EM signatures of both cell types were measured at UHF and the cell proportions in 100 MHz increments are shown (I). All results are represented as mean ± SEM except EM signatures represented as mean ± SD, NS *p*-value indicates lack of statistical significance, * *p*-value < 0.05, ** *p*-value < 0.01, *** *p*-value < 0.001 using one-way ANOVA test.

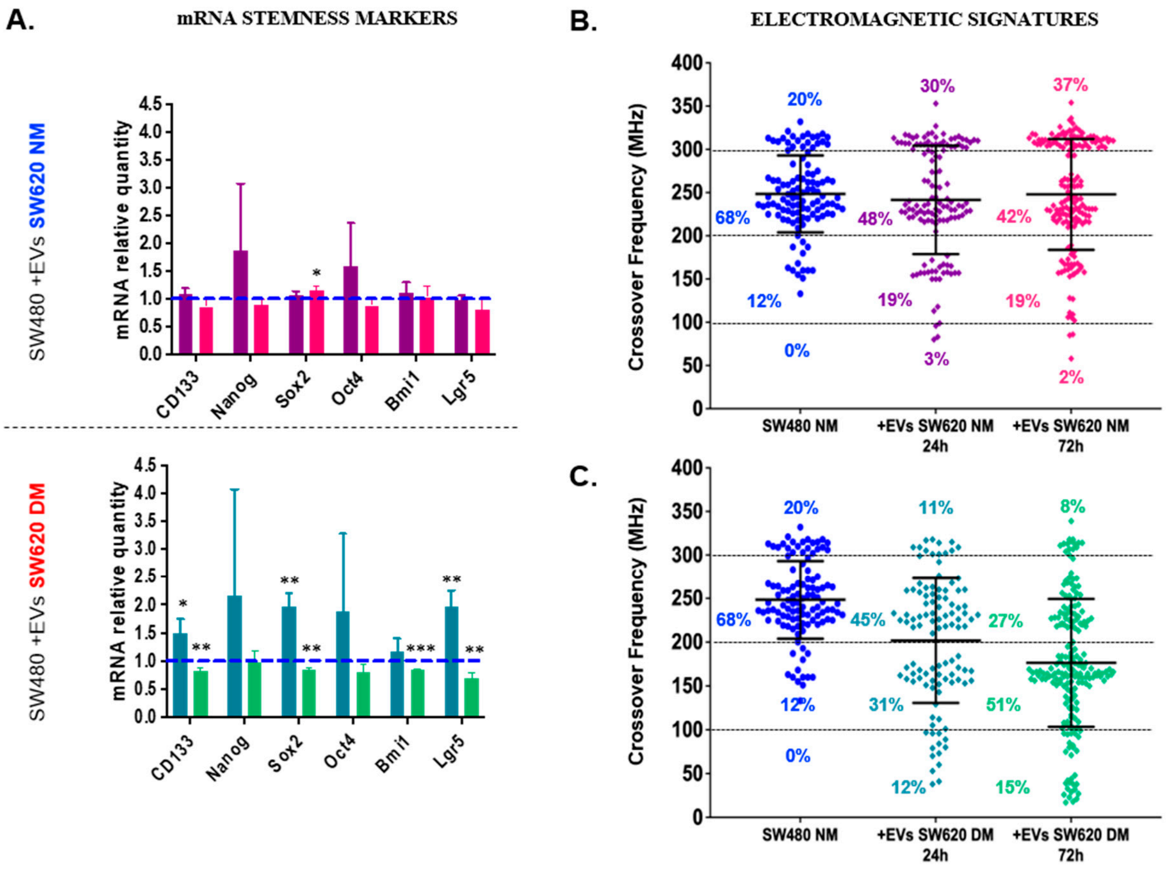

Figure S2. Effects of EVs derived from SW620-CSCs or differentiated cells on the SW480 cell line. SW480-NM cells were treated once for 24 h (purple and light blue conditions) or twice for 72h (pink and green conditions) with EVs derived from the SW620 cell line (NM or DM cultured cells). Stemness-related genes were analyzed according to treatment conditions (blue dotted line corresponding to untreated cells) (A). The EM signatures of cells treated with EVs derived from differentiated cells (B) or CSCs (C) were measured at UHF and the cell proportions in 100 MHz increments are shown. All results are represented as mean ± SEM except EM signatures represented as mean ± SD, NS *p*-value indicates lack of statistical significance, * *p*-value < 0.05, ** *p*-value < 0.01, *** *p*-value < 0.001 using one-way ANOVA test.

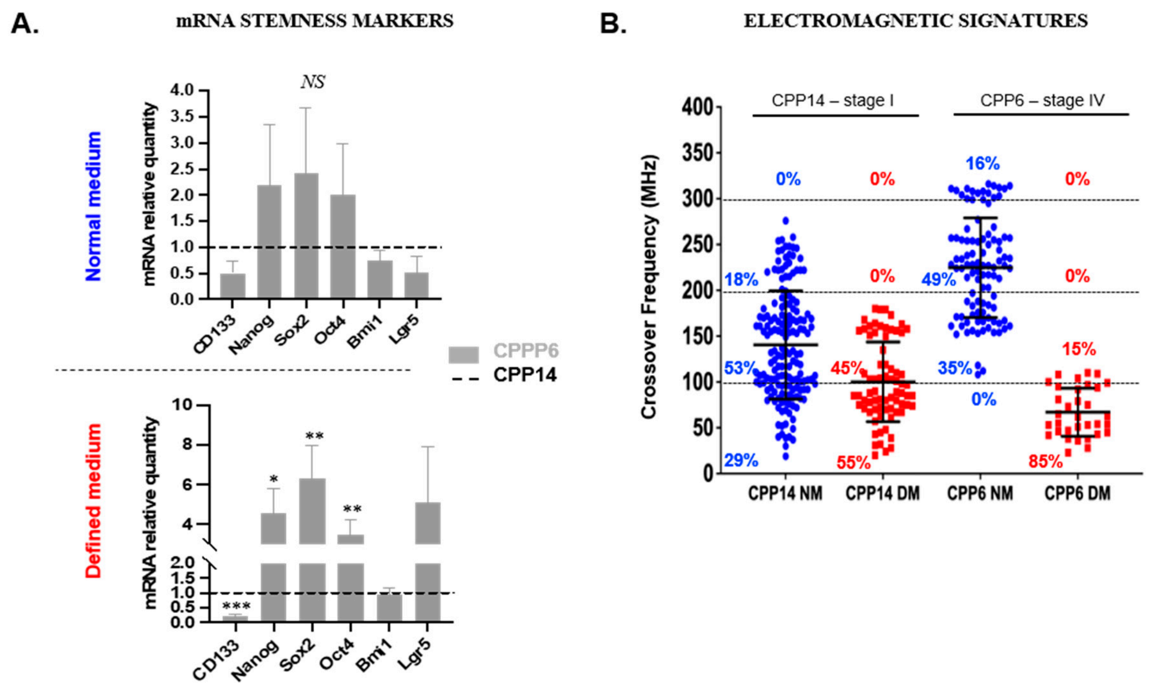

Figure S3. Characterization of patient-derived primary cells according to culture conditions. Two CRC patient-derived primary cultures (CPP14 and CPP6) were grown in normal (NM) or defined (DM) medium to enrich in differentiated cells or CSCs, respectively. Expression of stemness related genes was analyzed to compare both cell types (A). The EM signatures were measured at UHF according to culture conditions and the cell proportions in 100 MHz increments are shown (B). All results are represented as mean ± SEM except EM signatures represented as mean ± SD, NS *p*-value indicates a lack of statistical significance, * *p*-value < 0.05, ** *p*-value < 0.01, *** *p*-value < 0.001 using one-way ANOVA test.

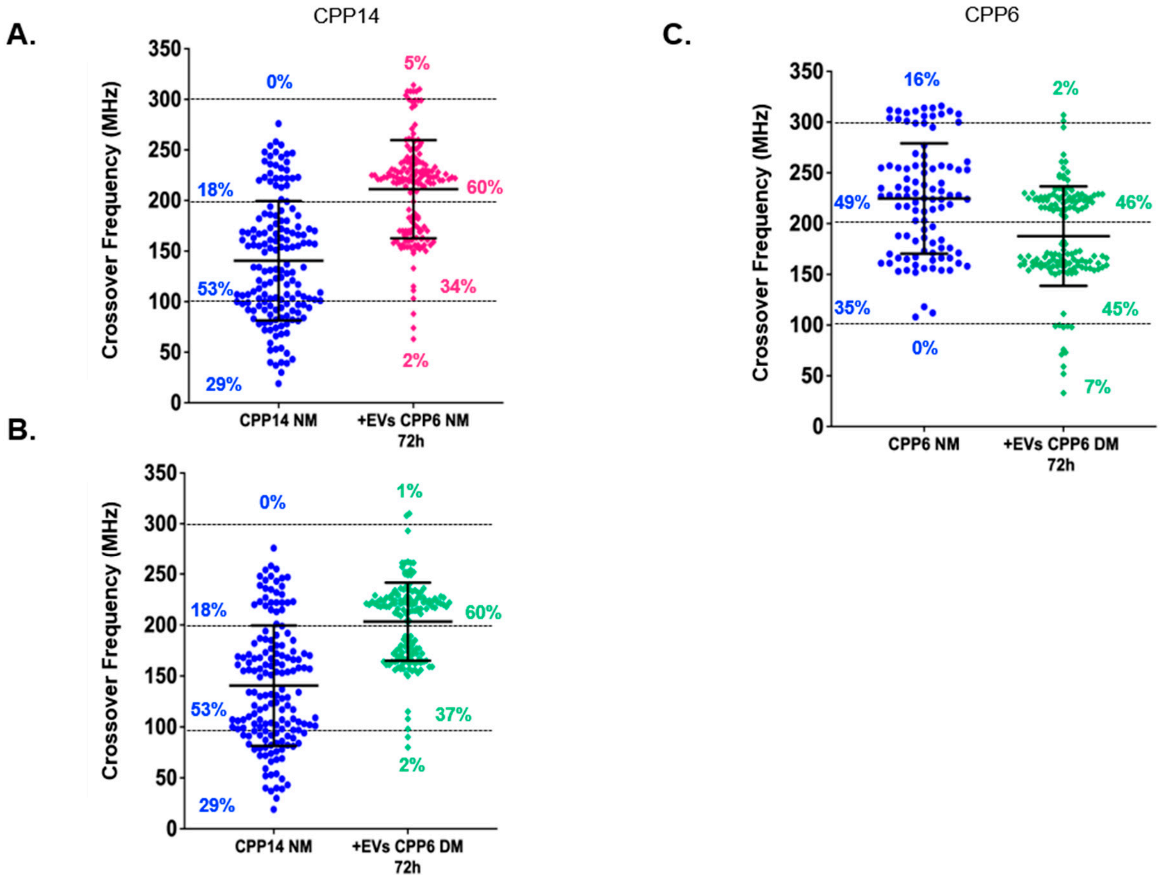

Figure S4. Effects of EVs derived from CPP6-CSCs or differentiated cells on the EM signatures of primary cells. CPP14-NM cells were treated twice for 72h with EVs derived from CPP6 primary cells, NM (A) or DM (B) cultured cells. CPP6-NM cells were treated twice for 72h with EVs derived from CPP6-DM cells (C). The EM signatures were measured at UHF and the cell proportions in 100 MHz increments are shown. All results are represented as mean ± SEM except EM signatures represented as mean ± SD, NS *p*-value indicates a lack of statistical significance, * *p*-value < 0.05, ** *p*-value < 0.01, *** *p*-value < 0.001 using one-way ANOVA test.

24 miRNA upregulated in EVs-treated cells as compared to control cells

Top-25 of most abundant miRNA detected in EVs

Target prediction of miRNA (miRDB, TargetScan, miRTARBase and DIANNAmicroT)

ENSEMBL ID	Gene Symbol	Gene ID	Number of predicted target				
			miRDB	Target Scan	DIANNA MicroT	miR TARBase	Common to 4 predicting tools
ENSG00000283880	MIR7704	102465802	64	2643	30	31	0
ENSG00000275110	NA	NA	0	0	0	0	0
ENSG00000277437	NA	NA	0	0	0	0	0
ENSG00000264063	NA	NA	0	0	0	0	0
ENSG00000263963	NA	NA	0	0	0	0	0
ENSG00000283813	MIR4485	100616263	118	2539	37	164	0
ENSG00000284154	MIR3605	100500853	517	4434	784	99	8
ENSG00000284419	MIR663A	724033	206	2522	91	111	4
ENSG00000266038	MIR4659A	100616348	2262	8262	5327	296	104
ENSG00000283386	MIR4659B	100616372	2317	8262	5457	295	112
ENSG00000278791	NA	NA	0	0	0	0	0
ENSG00000263675	MIR5581	100847010	487	6605	1492	123	9
ENSG00000284586	MIR92B	693235	1031	2048	1348	689	188
ENSG00000272036	MIR139	406931	785	3453	1113	201	27
ENSG00000207935	MIR204	406987	1834	6151	3033	520	112
ENSG00000221540	MIR1180	100302256	263	3490	267	105	3
ENSG00000274060	MIR6724-2	103504727	0	0	0	0	0
ENSG00000277379	MIR6724-3	103504739	0	0	0	0	0
ENSG00000275692	MIR6724-4	103504733	0	0	0	0	0
ENSG00000275950	MIR6724-1	102465433	266	3372	525	36	1
ENSG00000264607	MIR3173	100422981	1211	7411	2148	164	30
ENSG00000207611	MIR149	406941	2390	8000	2526	990	156
ENSG00000207571	MIR615	693200	323	3281	340	935	10
ENSG00000283867	MIR1307	100302174	12	1788	20	228	0

ENSEMBL ID	Gene Symbol	Gene ID	Number of predicted target				
			miRDB	Target Scan	DIANNA MicroT	miR TARBase	Common to 4 predicting tools
ENSG00000284190	MIR21	406991	1024	3925	1135	710	**114**
ENSG00000207864	MIR27B	407019	1716	3796	2558	467	**151**
ENSG00000284179	MIR7-1	407043	2037	5887	3124	169	**40**
ENSG00000207703	MIR7-2	407044	2034	898	3092	159	**10**
ENSG00000283484	MIR3529	100616238	1629	4893	1885	166	**39**
ENSG00000199072	MIRLET7F1	406888	1776	5244	1490	108	**35**
ENSG00000208012	MIRLET7F2	406889	1213	4756	1440	97	**26**
ENSG00000198973	MIR375	494324	271	304	1287	482	**19**
ENSG00000207808	MIR27A	407018	1713	4277	2546	465	**177**
ENSG00000199150	MIRLET7G	406890	1844	4883	2058	400	**135**
ENSG00000283990	MIRLET7A3	406883	1768	5244	2320	734	**91**
ENSG00000198975	MIRLET7A2	406882	957	4140	1187	87	**25**
ENSG00000207617	MIR3074	100422842	1150	7337	1546	162	**14**
ENSG00000284459	MIR24-1	407012	213	1923	228	28	**1**
ENSG00000284387	MIR24-2	407013	214	1923	232	27	**1**
ENSG00000283745	MIR196B	442920	415	718	584	166	**1**
ENSG00000283705	MIR92A1	407048	322	3286	336	66	**5**
ENSG00000199179	MIRLET7I	406891	1027	1577	1048	318	**91**
ENSG00000284538	MIR92A2	407049	1077	5872	1326	159	**36**
ENSG00000207759	MIR181A1	406995	1441	1897	2341	563	**168**
ENSG00000207595	MIR181A2	406954	517	4458	609	71	**4**
ENSG00000207635	MIR499A	574501	622	3500	2046	118	**9**
ENSG00000283441	MIR499B	100616134	1042	4259	1585	121	**11**
ENSG00000283867	MIR1307	100302174	12	1788	20	229	**0**
ENSG00000207924	MIR196A2	406973	925	4409	921	452	**47**

Functional Enrichment Analysis from commonly target genes of miRNA across four predicting tools (Gene Ontology-Biological Process, KEGG and Reactome)

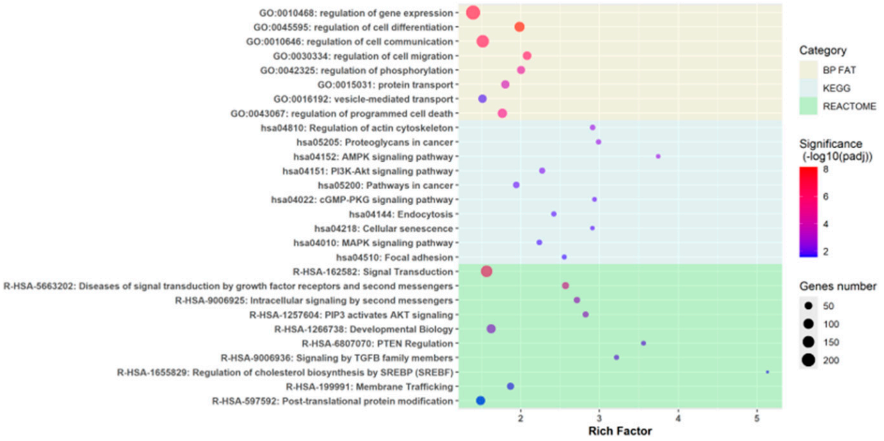

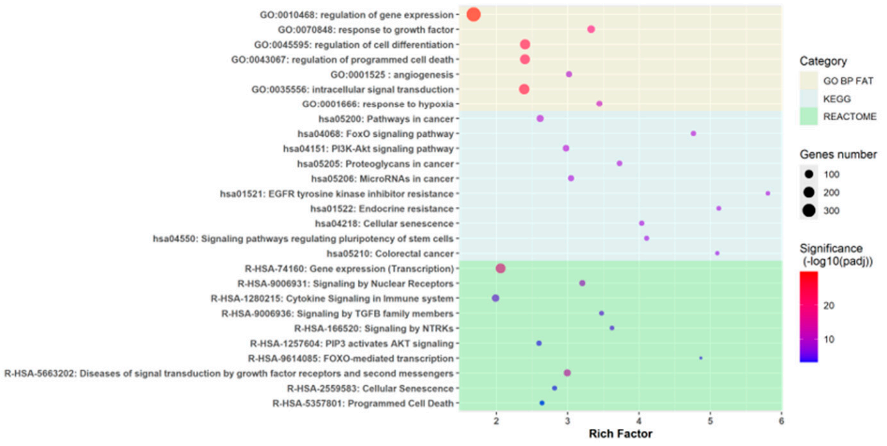

Figure S5. ***In silico*** **prediction of the biological effect of SW80 cells treatment with CSCs-derived EVs**

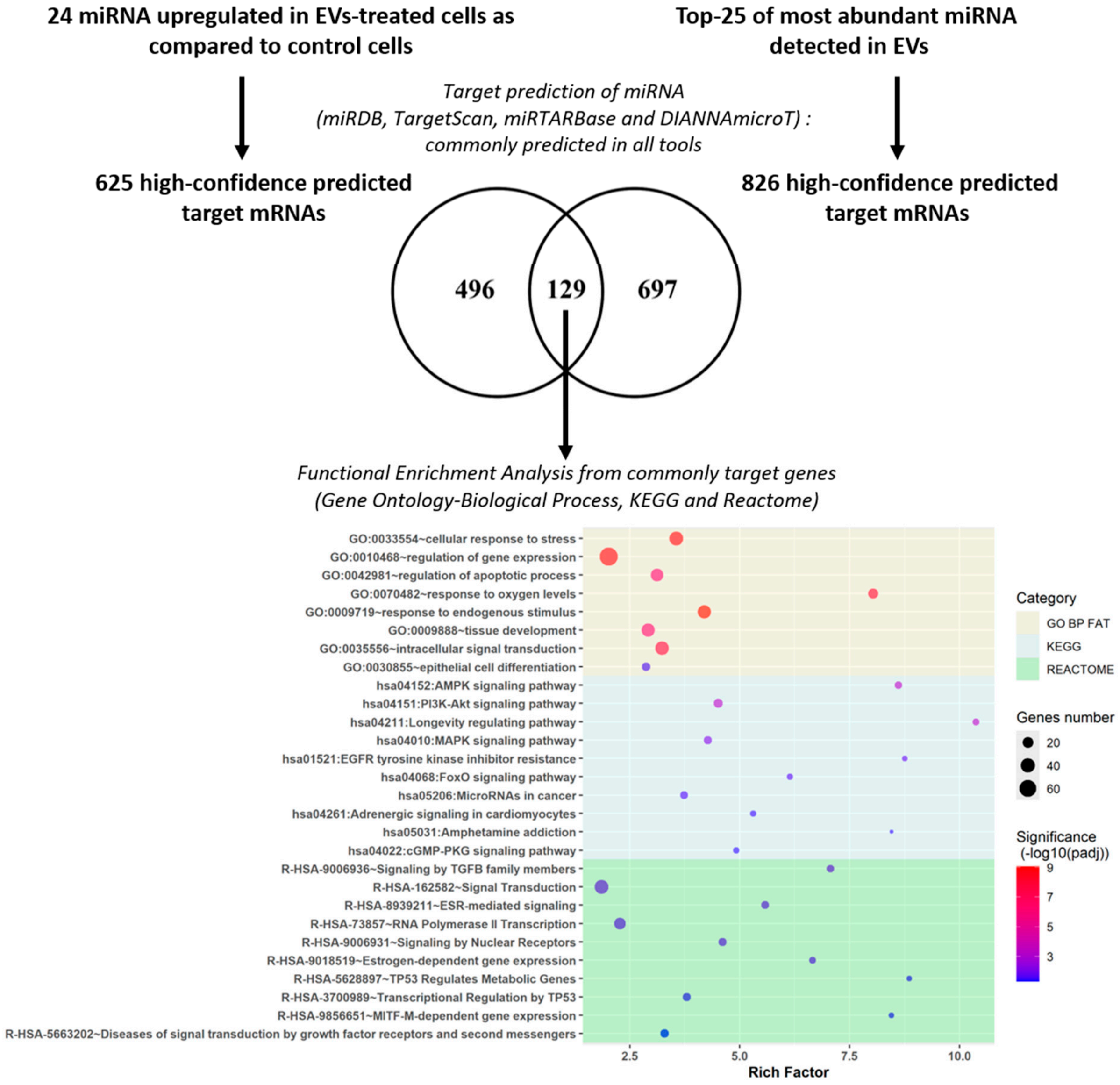

Figure S6. Functional Enrichment Analysis of genes commonly targeted by miRNAs detected in CSCs-derived EVs or deregulated in treated SW480 cells
